# Supplementary figures and images for: Antimicrobial and antibiofilm evaluation of thymol, sodium azide, and sodium lauryl sulfate against multidrug-resistant pathogens: An integrated experimental and computational study
Source: PLoS One. 2026 Apr 7;21(4):e0345977. doi: 10.1371/journal.pone.0345977 (PMC13056186; doi:10.1371/journal.pone.0345977)

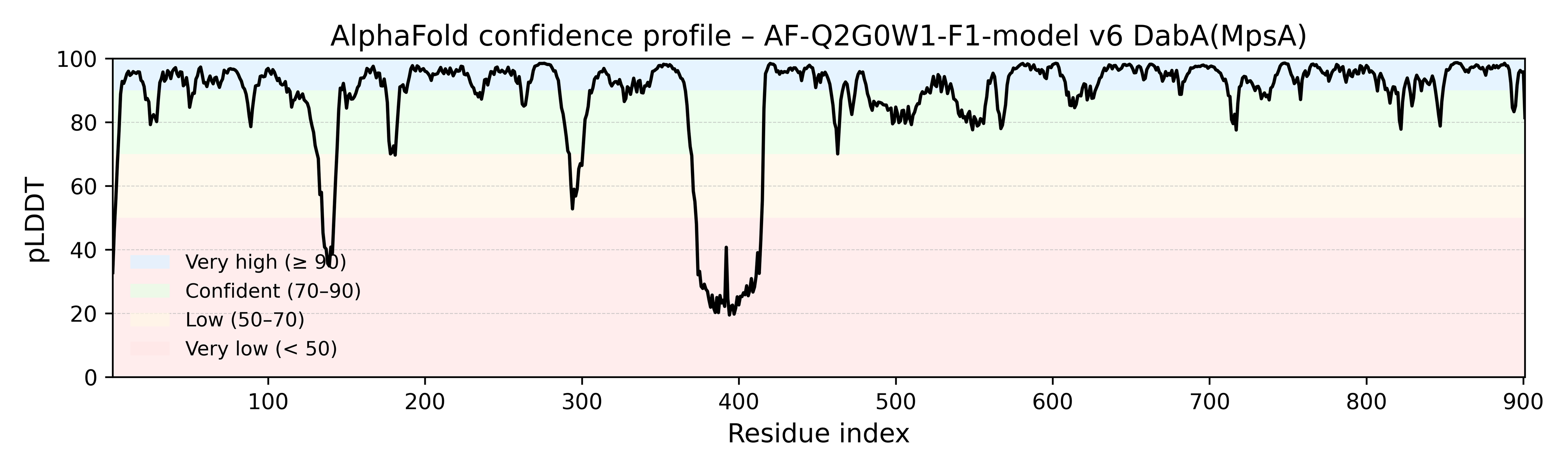

Supplement: S1 Fig — Per-residue pLDDT scores are plotted across the DabA sequence, with standard confidence ranges indicated (very high ≥90; high 70–90; low 50–70; very low < 50). The profile shows predominantly high-confidence regions, supporting a well-folded and structurally reliable model. (TIF) [file pone.0345977.s001.tif]

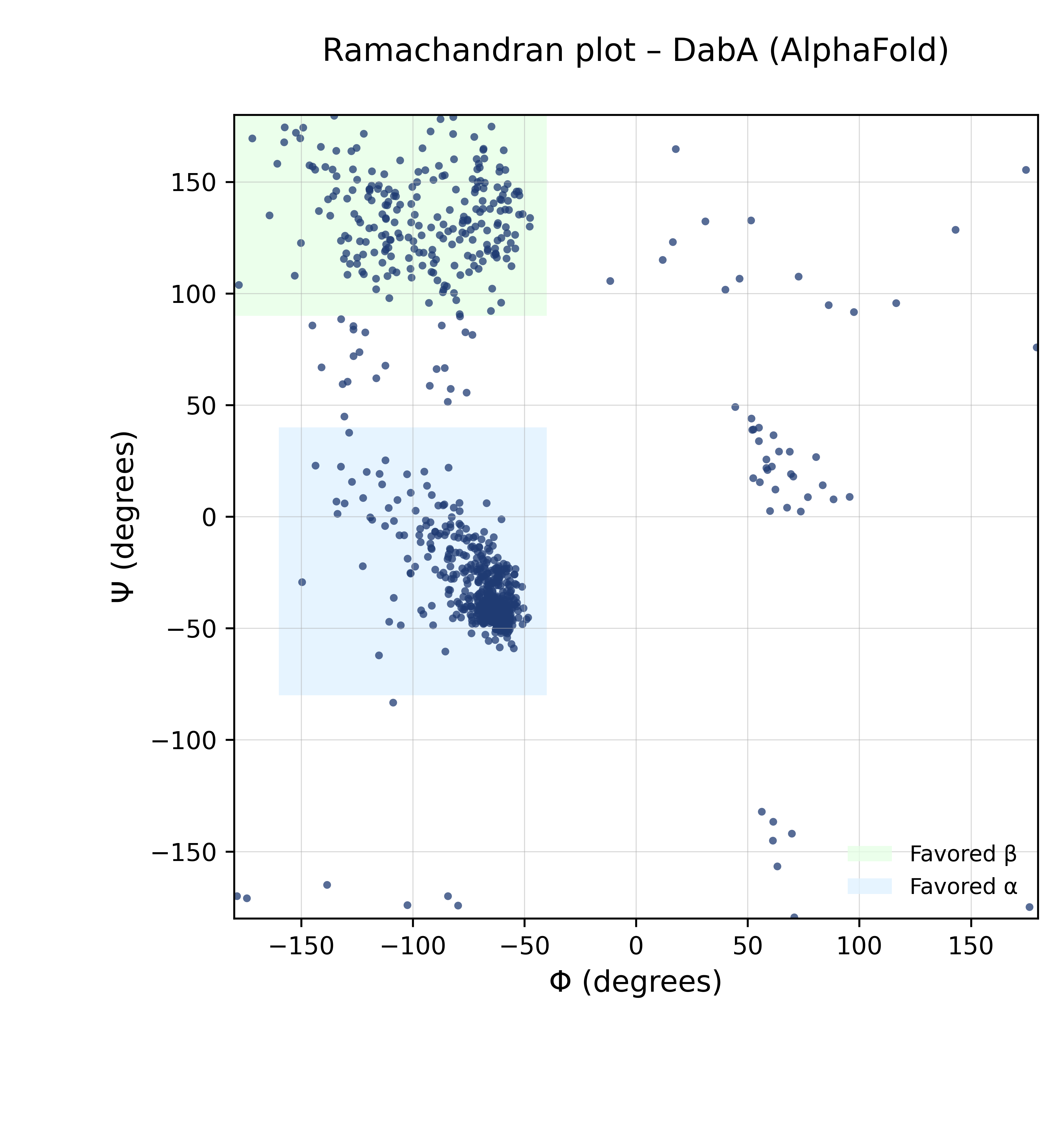

Supplement: S2 Fig — Backbone φ/ψ dihedral angles for all non-glycine and non-proline residues are displayed. Most residues fall within favored α-helical and β-sheet regions, indicating good stereochemical geometry and supporting the structural quality of the model. (TIF) [file pone.0345977.s002.tif]

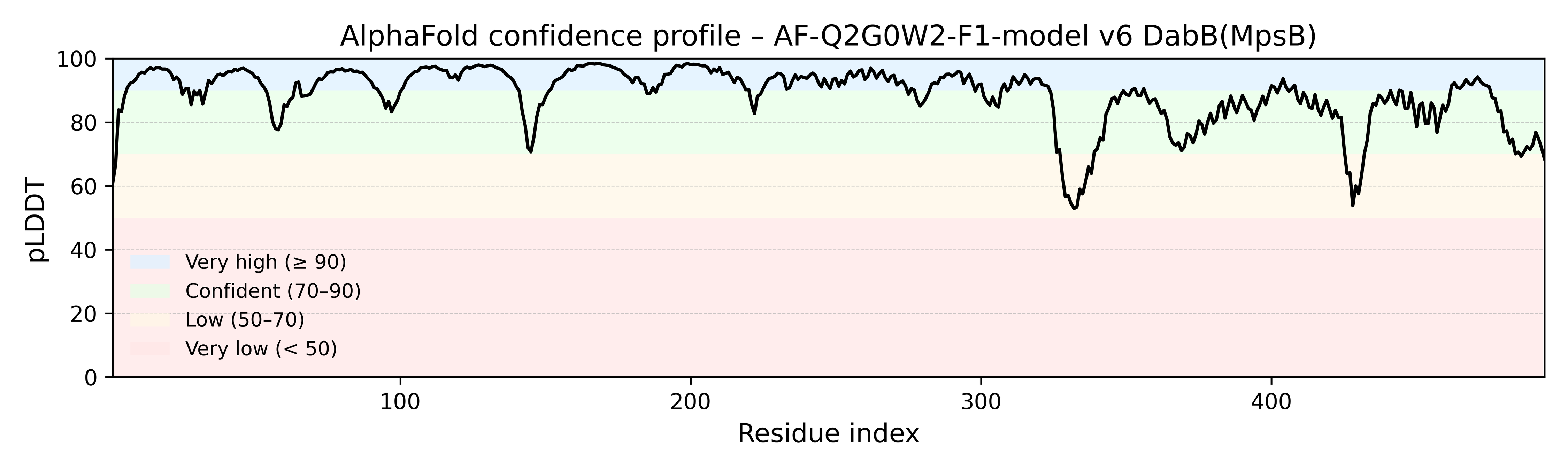

Supplement: S3 Fig — Residue-wise pLDDT values are shown with confidence zones marked. DabB exhibits consistently high-confidence predictions across structured domains, with moderate-confidence values limited to expected flexible or loop regions. (TIF) [file pone.0345977.s003.tif]

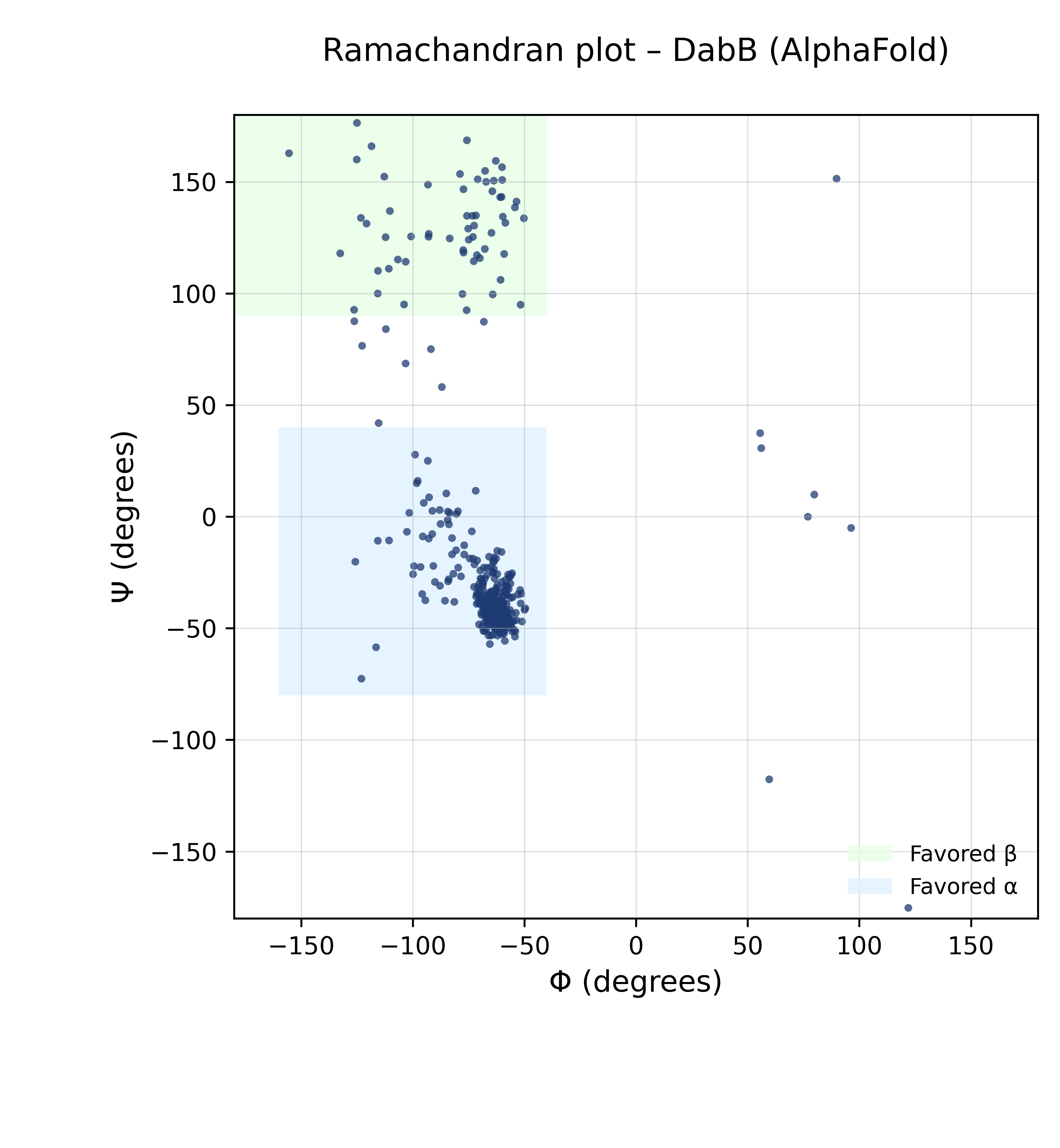

Supplement: S4 Fig — The φ/ψ dihedral angle distribution indicates that most residues occupy favored secondary-structure regions, demonstrating good stereochemical quality. Only a small number of residues fall in outlier regions, primarily within flexible loop segments. (TIF) [file pone.0345977.s004.tif]

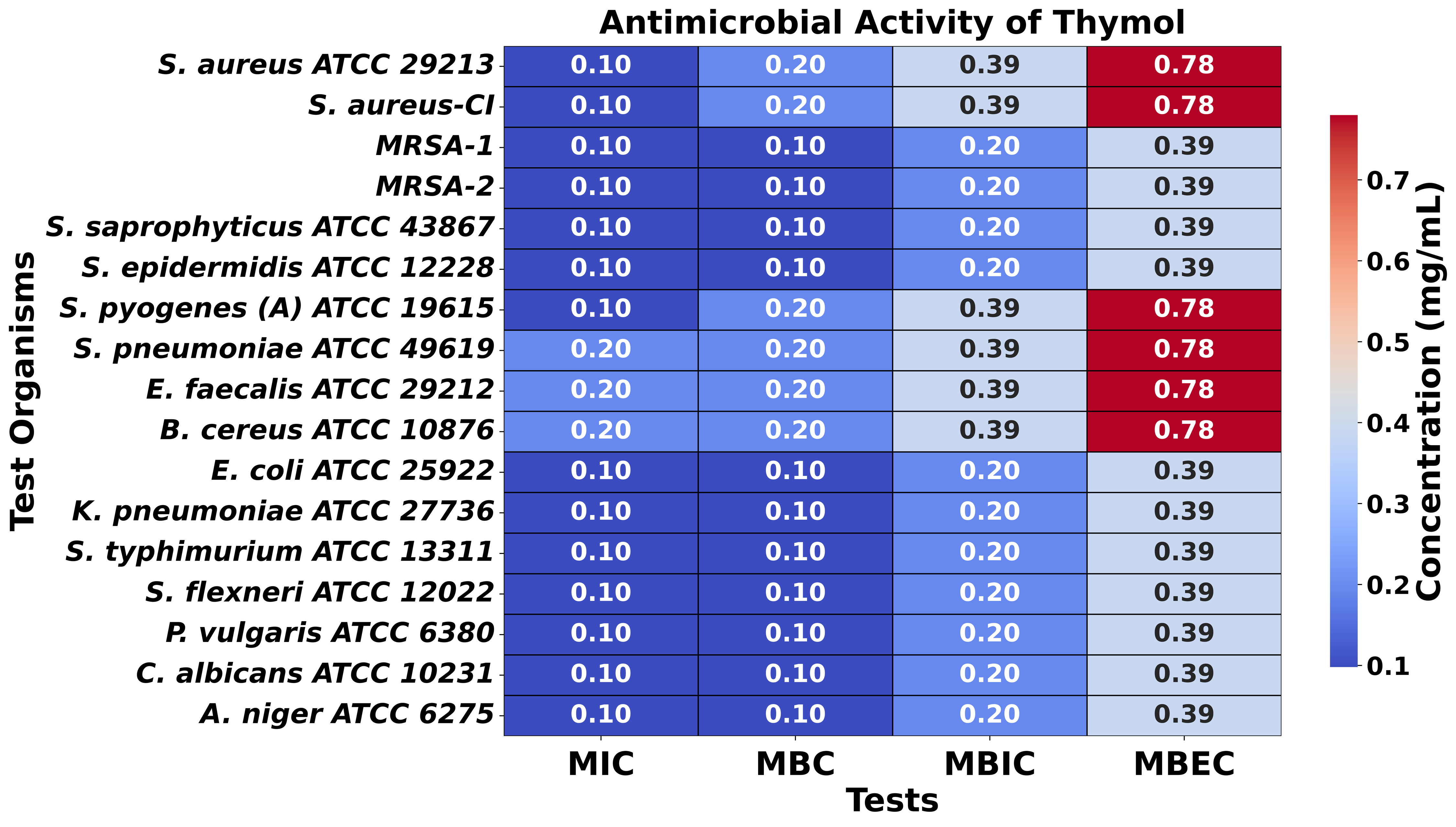

Supplement: S6 Fig — Heatmap showing MIC, MBC, MBIC, and MBEC values (mg/mL) of TM against the tested pathogens. Lower values indicate higher antimicrobial potency. All assays were performed in three independent biological replicates (n = 3); consensus values are reported. (TIF) [file pone.0345977.s006.tif]

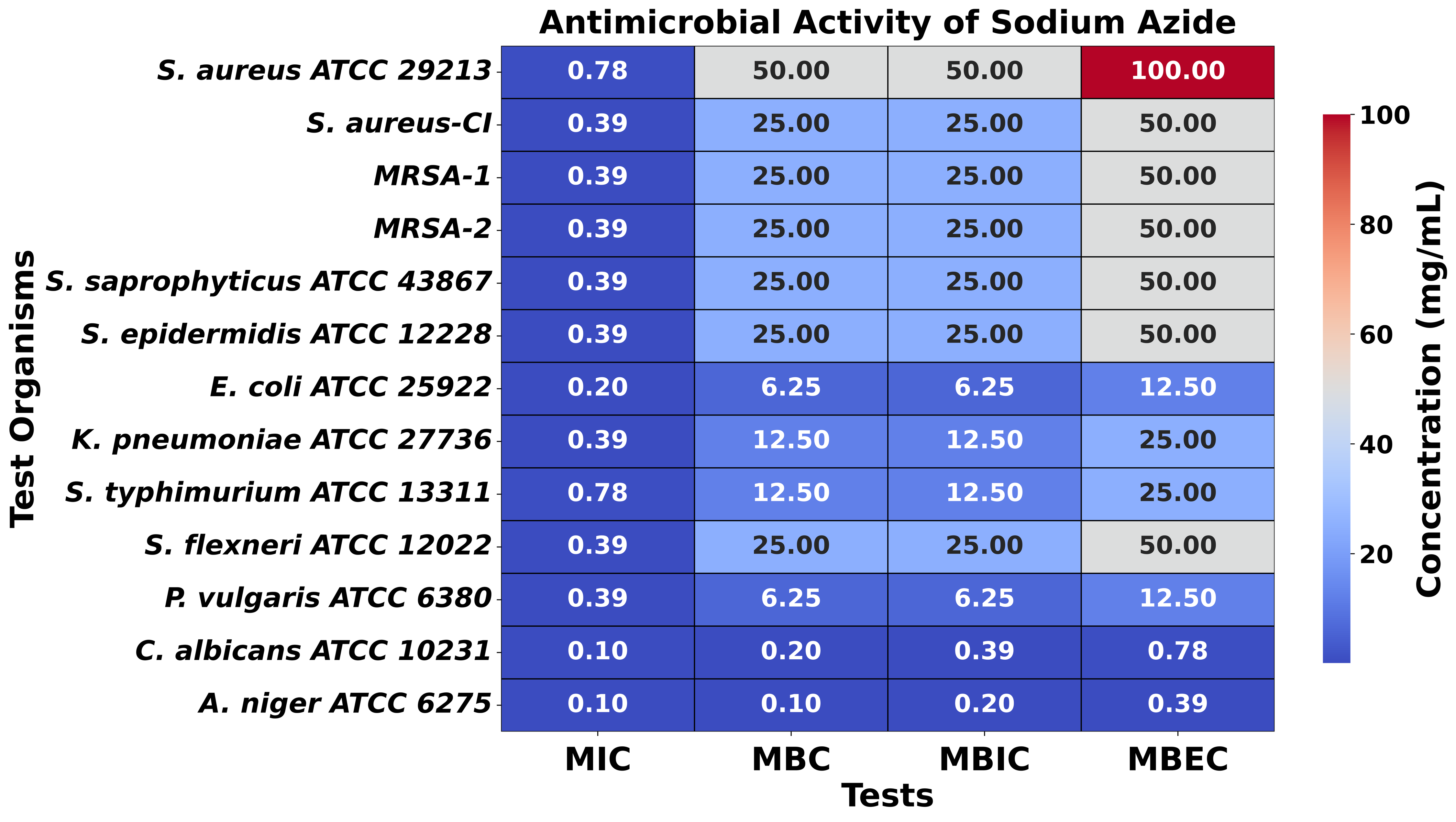

Supplement: S7 Fig — Heatmap showing MIC, MBC, MBIC, and MBEC values (mg/mL) of SA against the tested pathogens. Lower values indicate higher antimicrobial potency. All assays were performed in three independent biological replicates (n = 3); consensus values are reported. (TIF) [file pone.0345977.s007.tif]

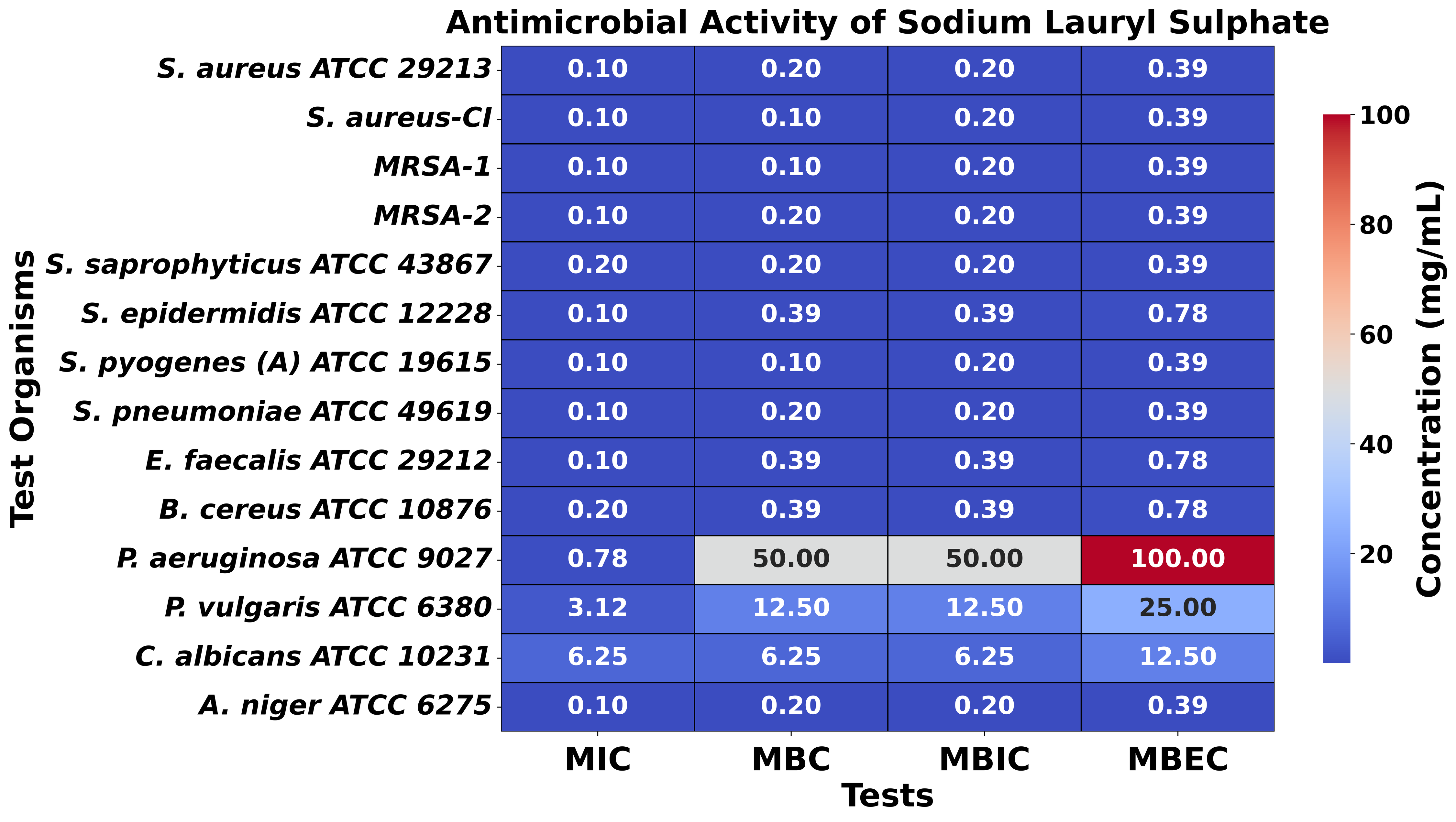

Supplement: S8 Fig — Heatmap showing MIC, MBC, MBIC, and MBEC values (mg/mL) of SLS against the tested pathogens. Lower values indicate higher antimicrobial potency. All assays were performed in three independent biological replicates (n = 3); consensus values are reported. (TIF) [file pone.0345977.s008.tif]

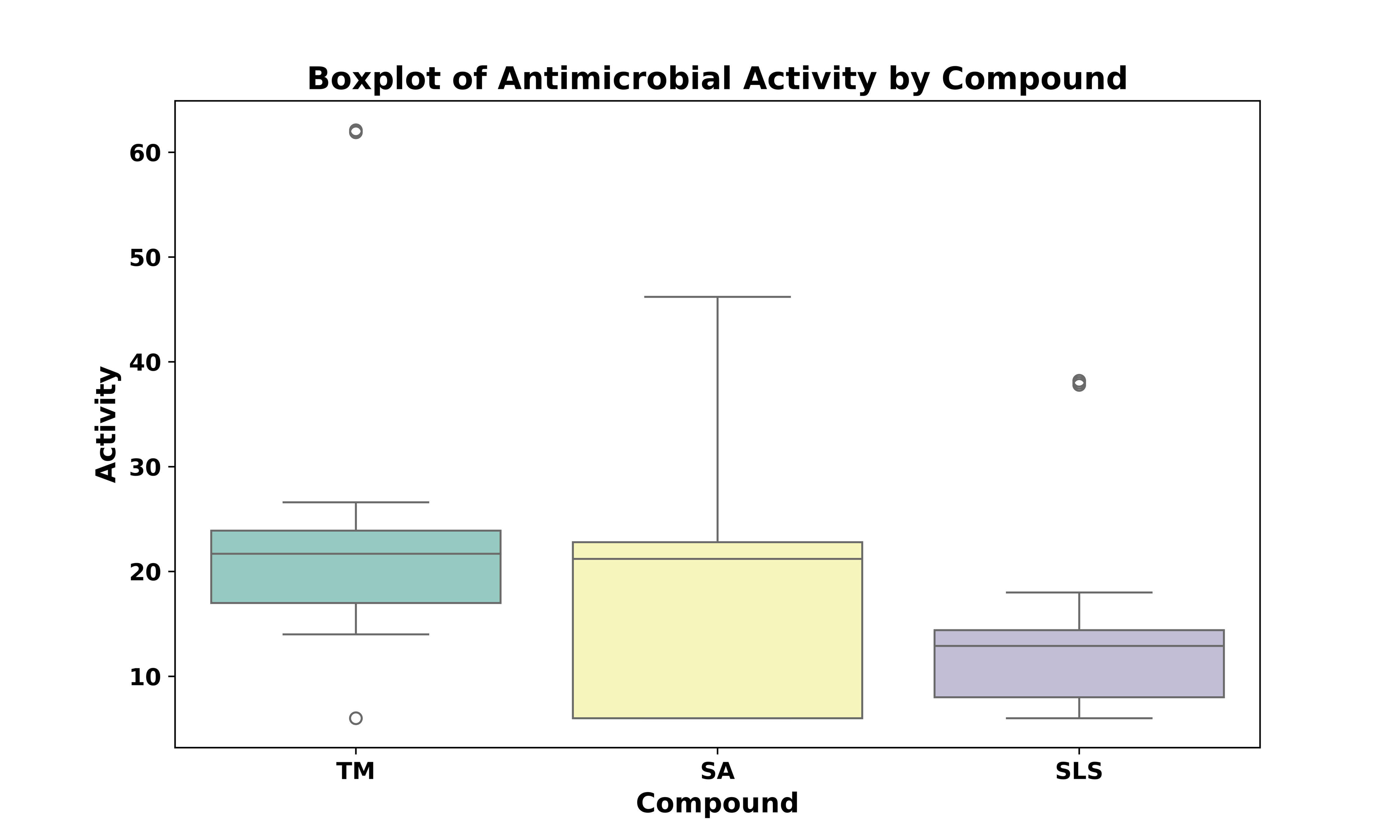

Supplement: S9 Fig — Box plots summarizing the distribution of inhibition zone diameters (mm) for TM, SA, and SLS across all tested pathogens. Statistical comparisons were performed using one-way ANOVA followed by Tukey’s HSD post-hoc test. Data represent three independent biological replicates (n = 3). (TIF) [file pone.0345977.s009.tif]

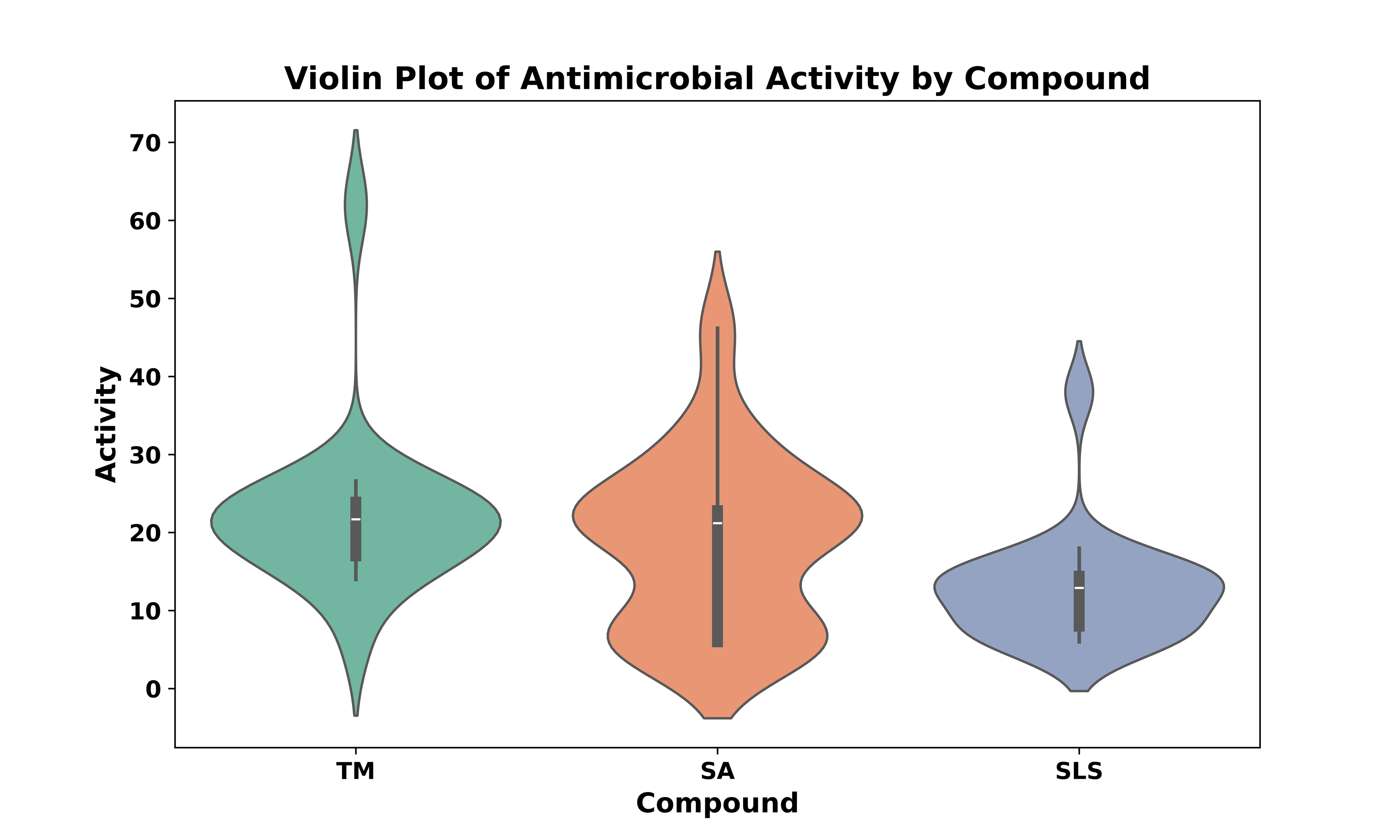

Supplement: S10 Fig — Violin plots illustrating the variation and density distribution of inhibition zone diameters (mm) for TM, SA, and SLS. Statistical analyses were performed using one-way ANOVA with Tukey’s HSD post-hoc test. Data represent three independent biological replicates (n = 3). (TIF) [file pone.0345977.s010.tif]

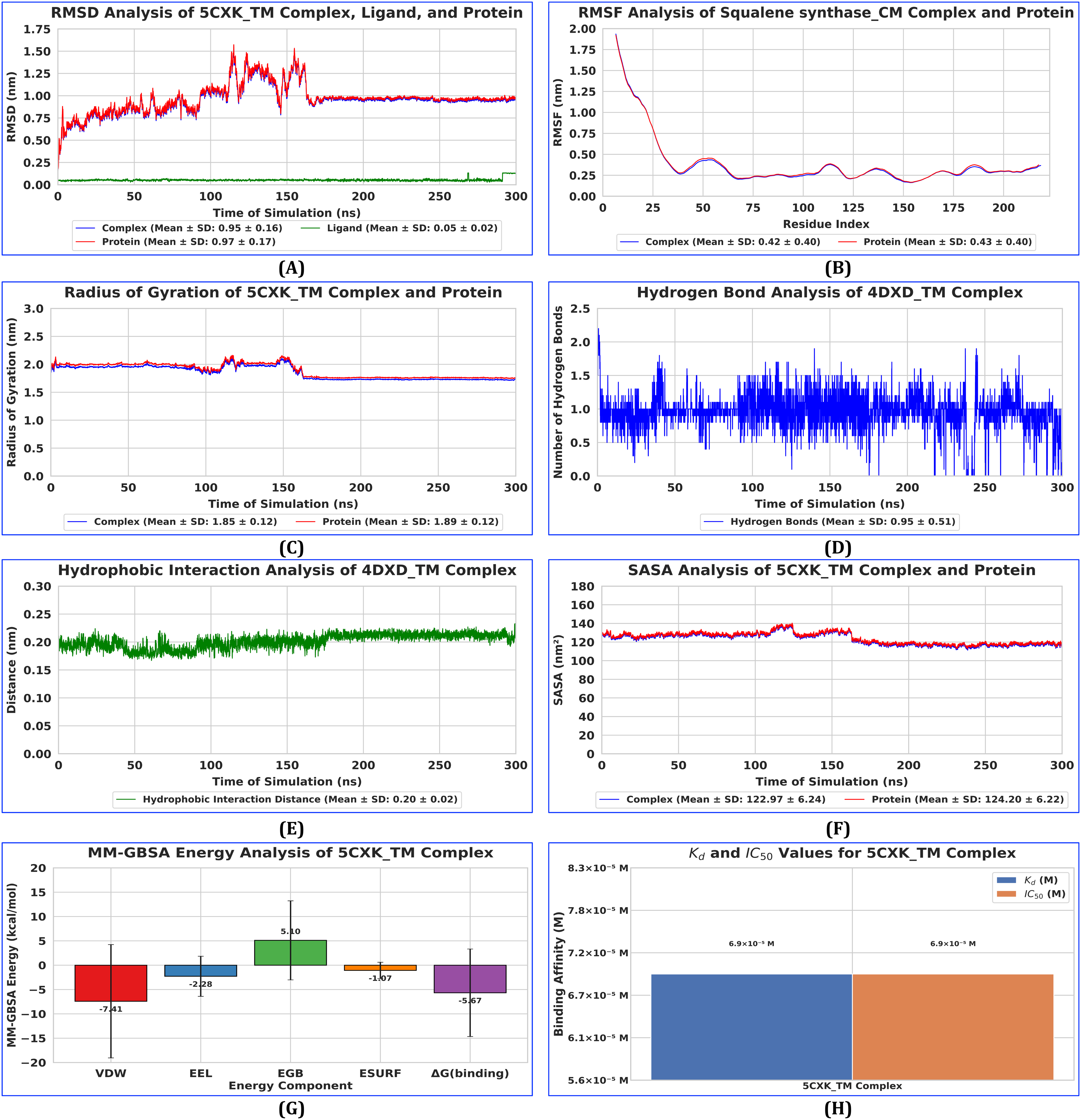

Supplement: S11 Fig — This figure presents RMSD, RMSF, radius of gyration (Rg), solvent-accessible surface area (SASA), hydrogen-bond profiles, and MM/GBSA binding free energy (ΔG) for the 5CXK–TM complex. Estimated Kd and IC₅₀ values derived from ΔG are also included, summarizing overall complex stability and binding strength across 300 ns. (TIF) [file pone.0345977.s011.tif]

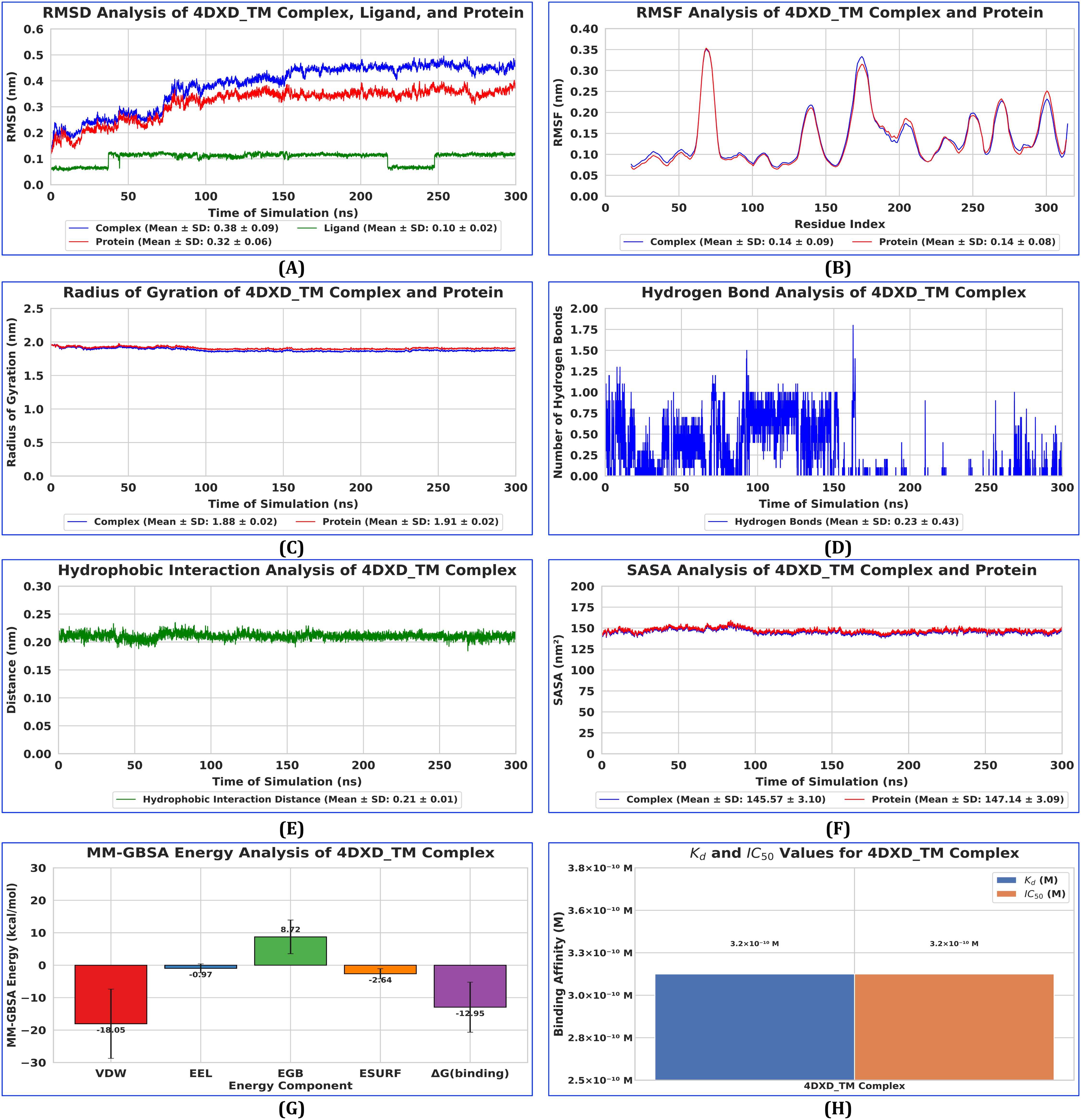

Supplement: S12 Fig — RMSD, RMSF, Rg, SASA, hydrogen-bond profiles, and MM/GBSA binding free energy (ΔG) for the 4DXD–TM complex are shown, along with estimated Kd and IC₅₀ values, providing a summary of structural stability and binding behavior over 300 ns. (TIF) [file pone.0345977.s012.tif]

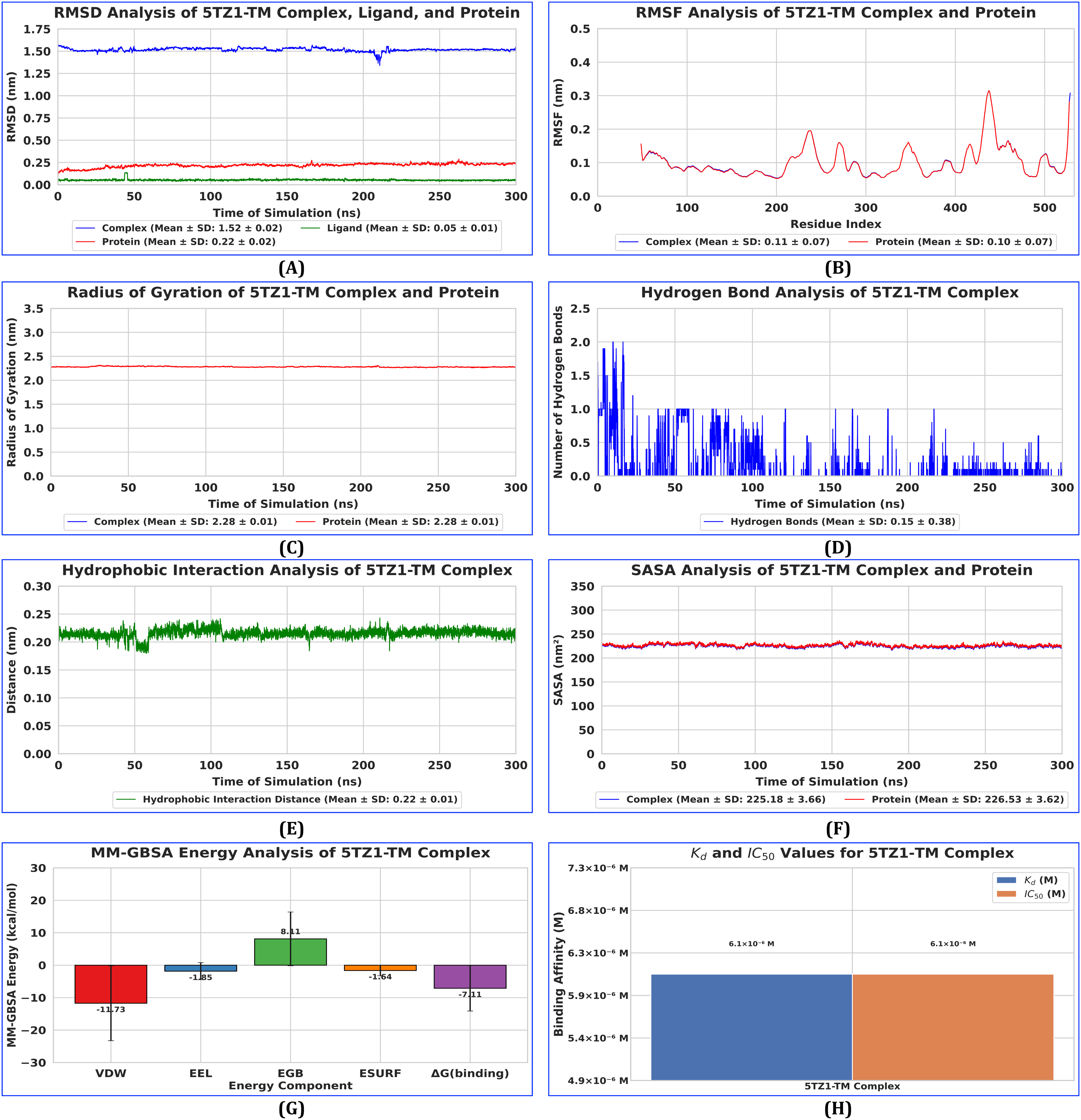

Supplement: S13 Fig — This figure includes RMSD, RMSF, Rg, SASA, hydrogen-bond patterns, and MM/GBSA binding free energy (ΔG) for the 5TZ1–TM complex, with corresponding Kd and IC₅₀ estimates reflecting overall stability and binding affinity across 300 ns. (TIF) [file pone.0345977.s013.tif]

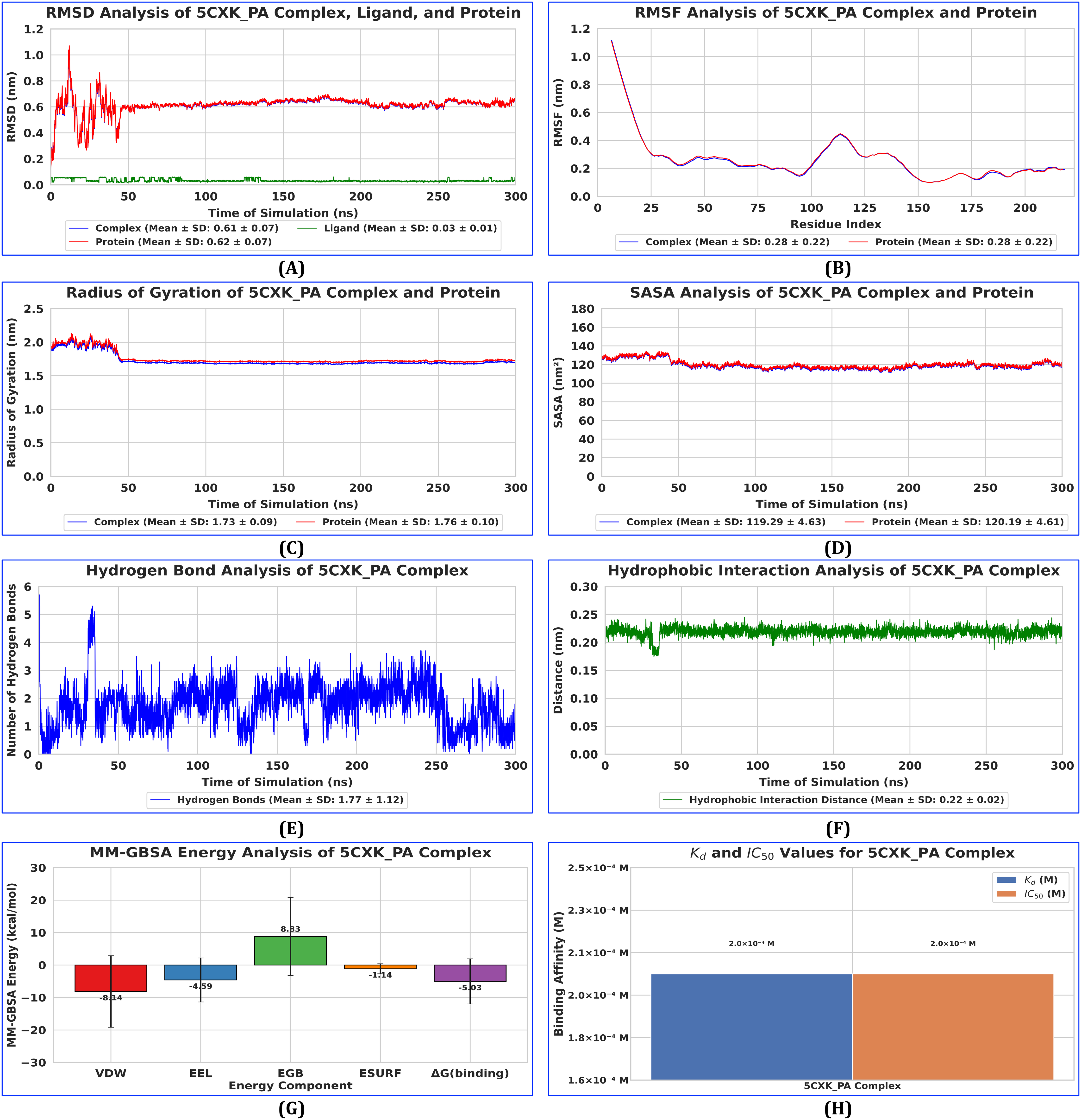

Supplement: S14 Fig — RMSD, RMSF, Rg, SASA, hydrogen-bond profiles, and MM/GBSA binding free energy (ΔG) for the 5CXK–PA complex are presented, together with estimated Kd and IC₅₀ values that summarize binding strength and structural stability throughout 300 ns. (TIF) [file pone.0345977.s014.tif]

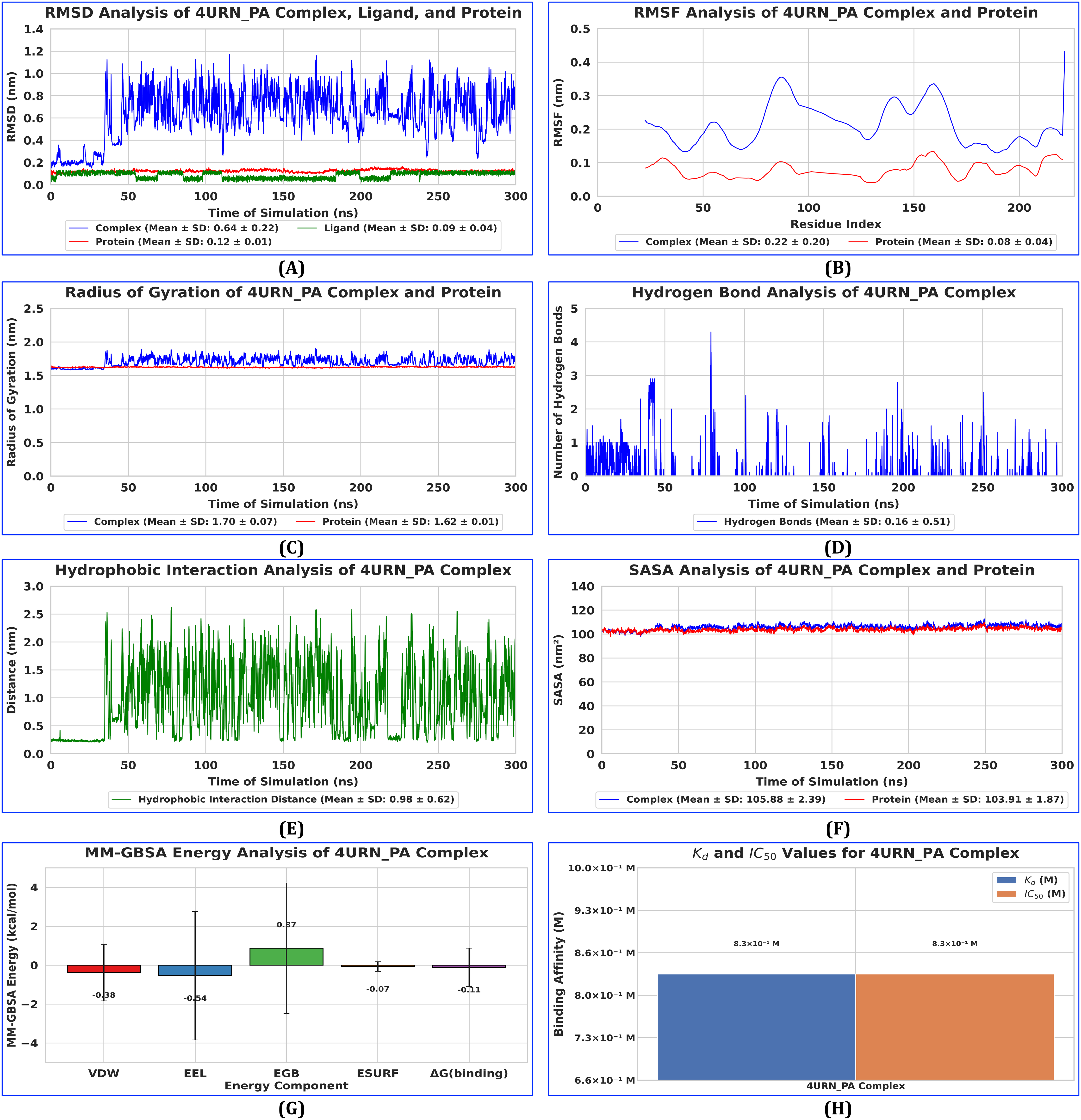

Supplement: S15 Fig — This figure reports RMSD, RMSF, Rg, SASA, hydrogen-bond data, and MM/GBSA binding free energy (ΔG) for the 4URN–PA complex, along with derived Kd and IC₅₀ values, providing insight into complex stability over 300 ns. (TIF) [file pone.0345977.s015.tif]

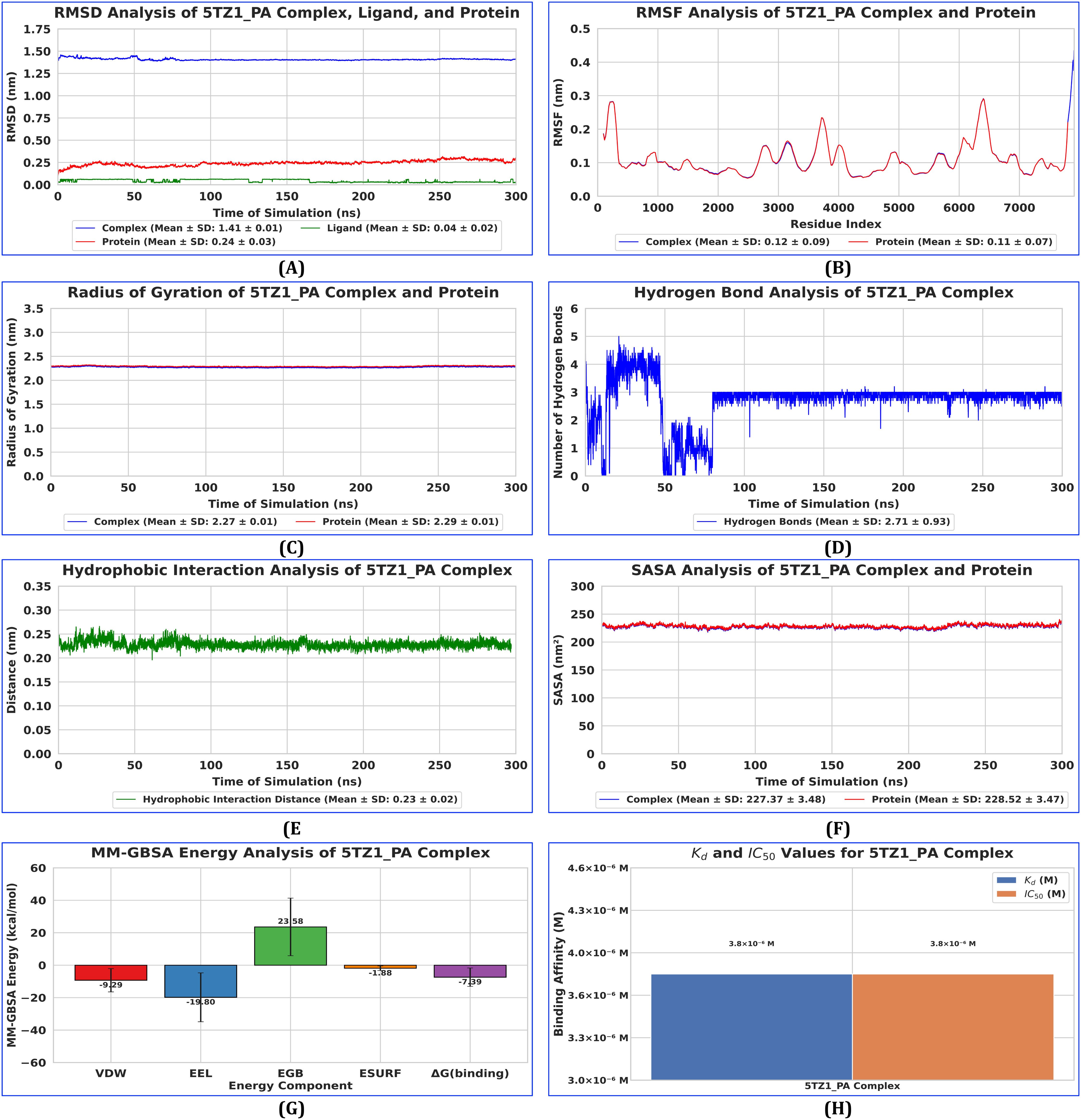

Supplement: S16 Fig — RMSD, RMSF, Rg, SASA, hydrogen-bond profiles, and MM/GBSA binding free energy (ΔG) are shown for the 5TZ1–PA complex. Estimated Kd and IC₅₀ values further summarize binding affinity and stability during 300 ns. (TIF) [file pone.0345977.s016.tif]

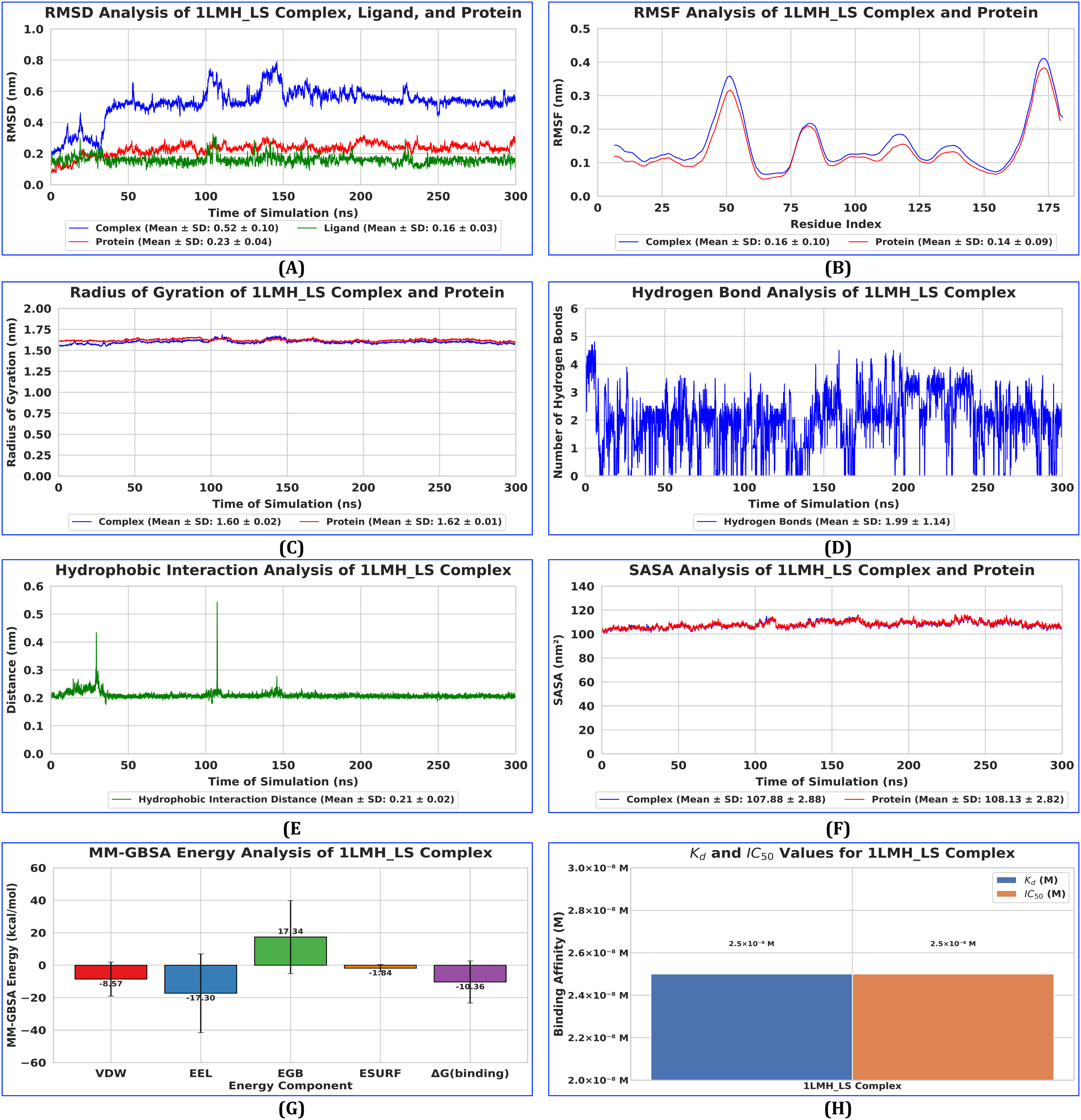

Supplement: S17 Fig — This figure presents RMSD, RMSF, Rg, SASA, hydrogen-bond behavior, and MM/GBSA binding free energy (ΔG) for the 1LMH–LS complex, with estimated Kd and IC₅₀ values indicating overall complex stability and binding strength across 300 ns. (TIF) [file pone.0345977.s017.tif]

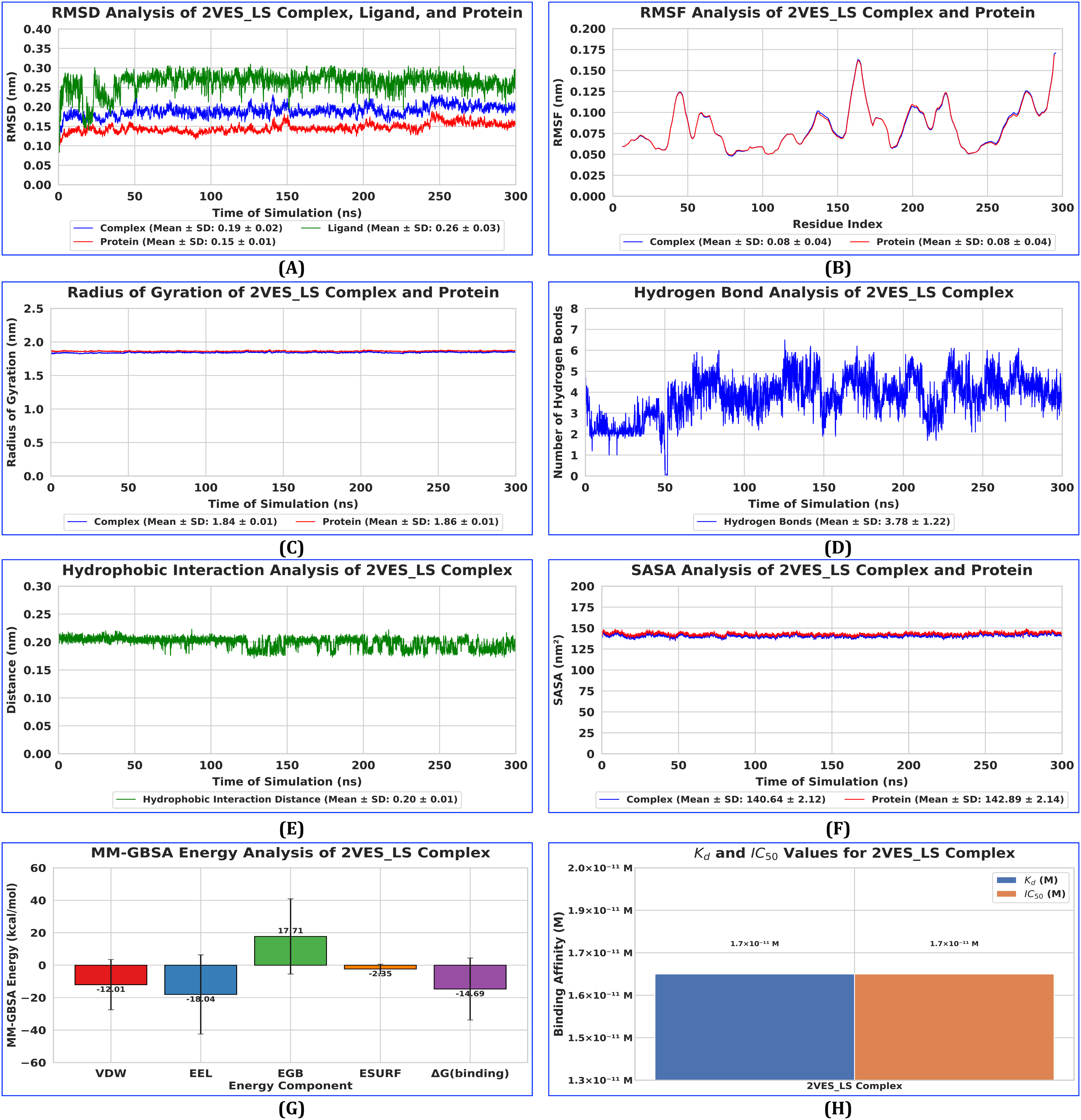

Supplement: S18 Fig — RMSD, RMSF, Rg, SASA, hydrogen-bond profiles, and MM/GBSA binding free energy (ΔG) for the 2VES–LS complex are included, along with Kd and IC₅₀ estimates summarizing stability and binding interactions during the whole 300 ns simulation. (TIF) [file pone.0345977.s018.tif]

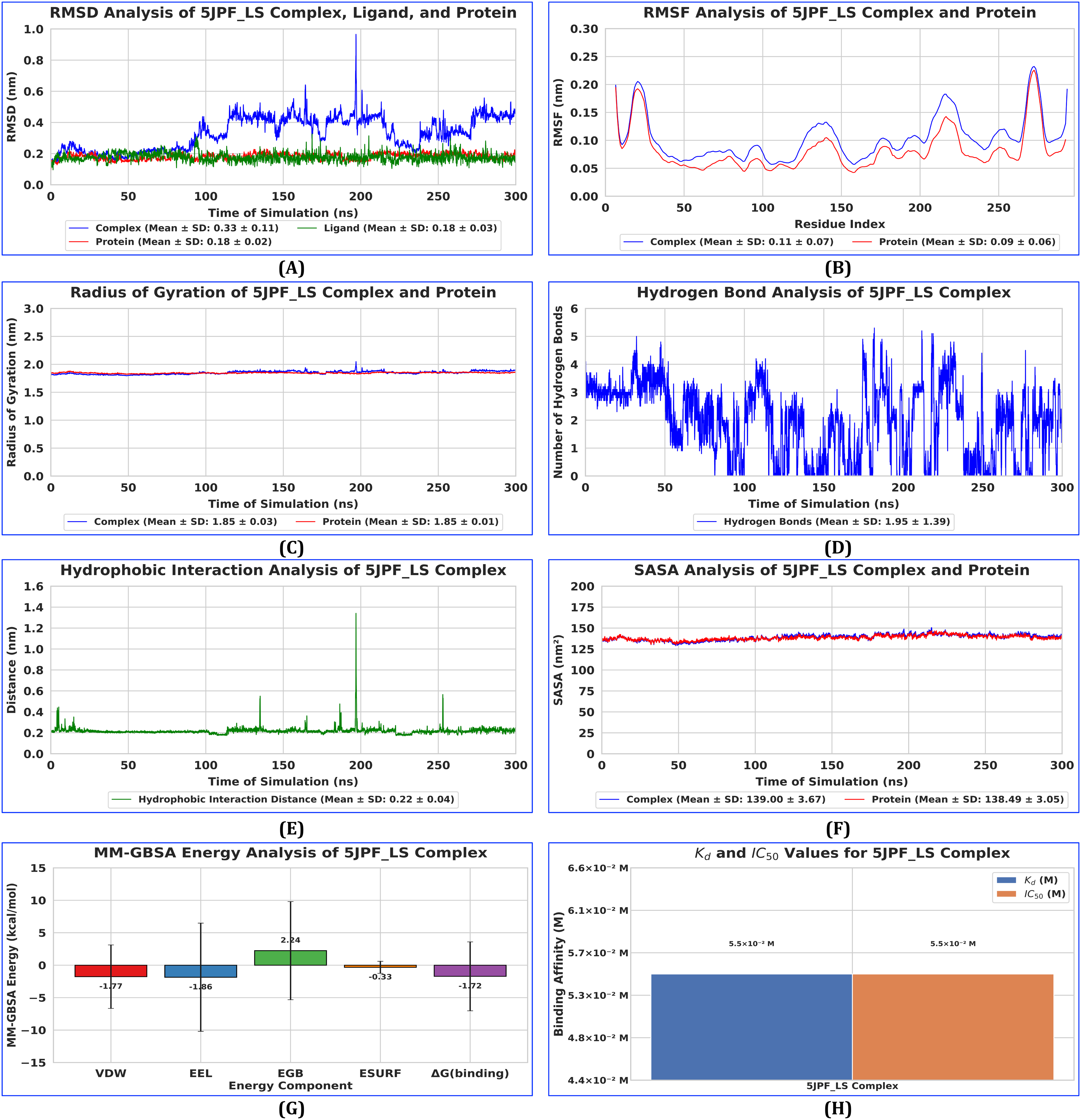

Supplement: S19 Fig — This figure shows RMSD, RMSF, Rg, SASA, hydrogen-bond trends, and MM/GBSA binding free energy (ΔG) for the 5JPF–LS complex. Estimated Kd and IC₅₀ values provide an overview of binding affinity and structural stability over 300 ns. (TIF) [file pone.0345977.s019.tif]

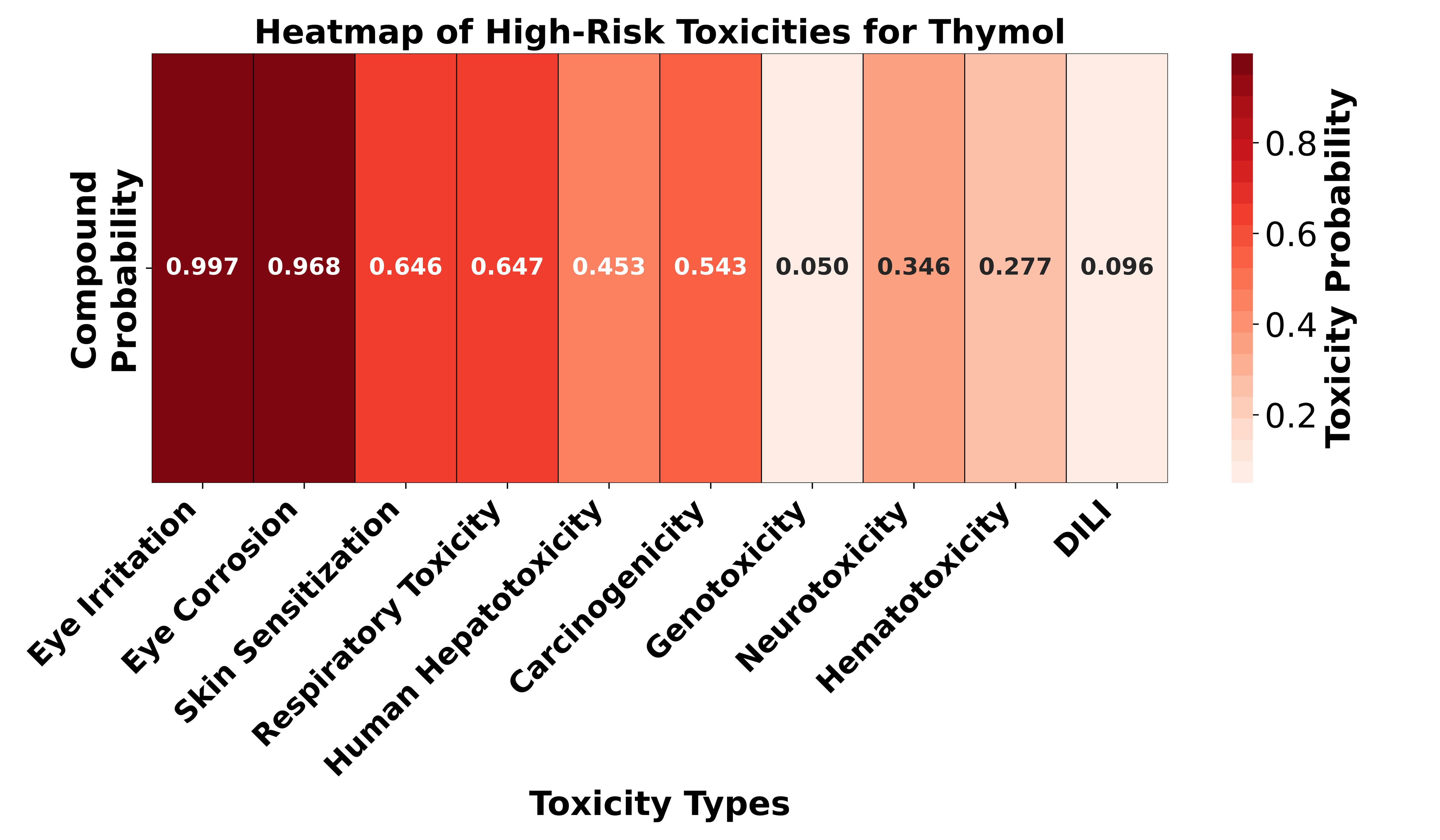

Supplement: S29 Fig — Heatmap showing predicted probabilities of hepatotoxicity, carcinogenicity, genotoxicity, neurotoxicity, and nephrotoxicity for TM. Dark red indicates higher predicted toxicity (values approaching 1), while lighter shades indicate lower risk. (TIF) [file pone.0345977.s029.tif]

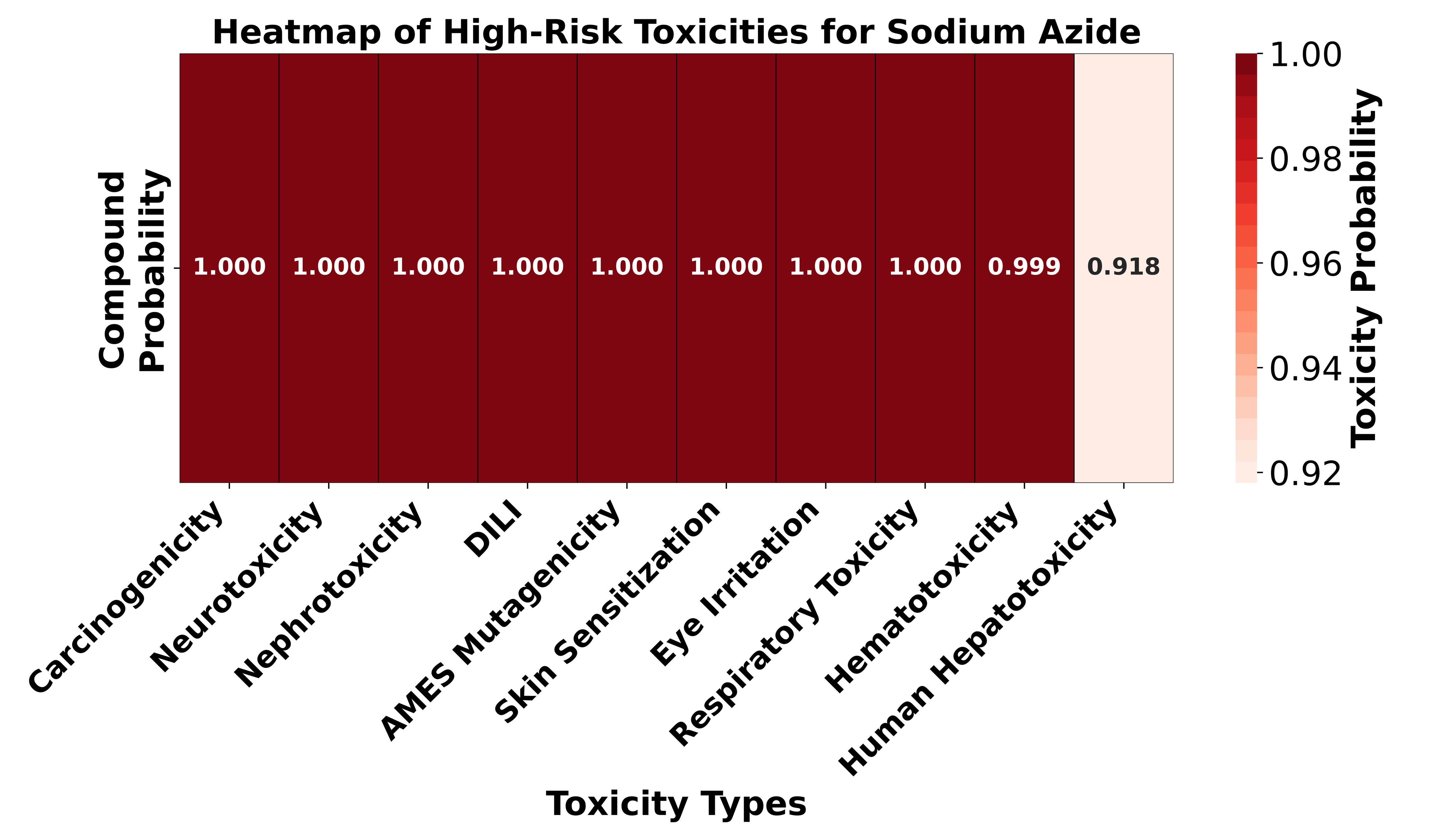

Supplement: S30 Fig — Heatmap illustrating predicted probabilities of toxicity across hepatotoxicity, carcinogenicity, genotoxicity, neurotoxicity, and nephrotoxicity for SA. Color intensity reflects the likelihood of adverse effects. (TIF) [file pone.0345977.s030.tif]

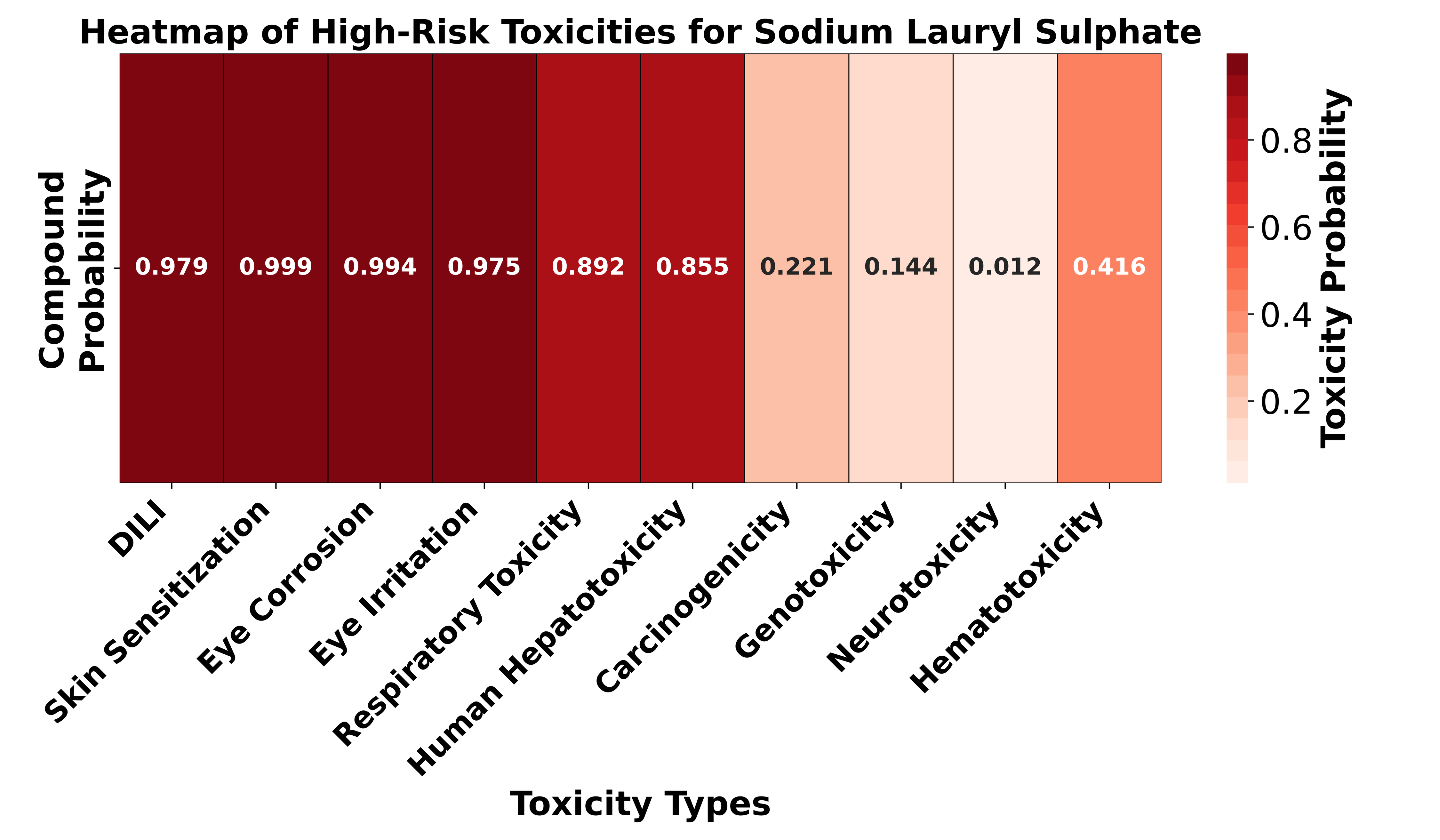

Supplement: S31 Fig — Heatmap displaying predicted toxicity probabilities for SLS across hepatotoxicity, carcinogenicity, genotoxicity, neurotoxicity, and nephrotoxicity. A darker color corresponds to a higher predicted risk. (TIF) [file pone.0345977.s031.tif]
