# Supplementary material for: Antimicrobial and antibiofilm evaluation of thymol, sodium azide, and sodium lauryl sulfate against multidrug-resistant pathogens: An integrated experimental and computational study
Source: PLoS One. 2026 Apr 7;21(4):e0345977. doi: 10.1371/journal.pone.0345977 (PMC13056186; doi:10.1371/journal.pone.0345977)
Supplement: S1 Table — This table lists the microbial proteins screened against thymol (TM), phenyl azide (PA; analog of sodium azide [SA]), and lauryl sulfate (LS; an analog of sodium lauryl sulfate [SLS]). For each target, the enzyme name, source organism, FASTA sequence, amino acid length, structural information (PDB code or model source), and associated biological pathway are provided. These proteins represent essential bacterial and fungal pathways and were selected to enable a comprehensive evaluation of ligand–protein interactions. (DOCX) [file pone.0345977.s032.docx]

| **#** | **Enzyme name** | **Source organism** | **FASTA**  **sequence** | **Amino acid length** | **PDB code or model source** | **Biological role** |
| --- | --- | --- | --- | --- | --- | --- |
| 1. | DabA (MpsA) | *Staphylococcus aureus* strain NCTC 8325 | MTTQLNINSVIENAKRVITPLSPISIFAARNPWEGLEADTFEDVAKWLRDVRDVDIFPNKALIESAVARGELDESVFNQLVTDMLLEHHYNIPQHYINLYIDNIKTLKDVPASYMNHSNVDVVADLLLEKSKRDMAESYHHYDVRPMSDAIIDEQGEPLSEQVNRQMIKWTKLYIDQFLSSWTMPKREQSFYHAWLHLAQHDHSFTKAQRQVIKGLPNDPEMTIESVLTHFSIDQEDYQAYVEGHLLALPGWAGMLYYRSQQHHFEQHLLTDYLAIRLVVEQLLVGDEFKSVAKDCESRSENWFKQTVASWCYYSDMPSDVLLQHDVNEIQTFIHFAATMNKNVFKNLWLIAWEMTYESQLKQKIKAGHESVAGALDVNQVNVSENDNANQPHSVLLNDTQAVDENNSELNQMGTSTKAQIAFCIDVRSEPFRRHIEAAGPFETIGIAGFFGLPIQKDAVDEQFKHDSLPVMVPPAYRIKEFADRYDMNVYRQQQQTMSSMFYTFKLMKNNVMPSLLLPELSGPFLSLSTIVNSIMPRKSRASLQKIKQKWLKKPETKLTIDREFDRTSDLPVGFTEQEQIDFALQALKLMDLTEAFAPFVVLAGHASHSHNNPHHASLECGACGGASSGFNAKLLAMICNRPNVRQGLKQSGVYIPETTVFAVAEHHTSTDTLAWVYVPDTLSSIALDAYESLNDAMPMISEHANRERLDKLPTIGRVNHPVEEAQRFASDWSEVRPEWGLAKNASFIIGRRQLTKGIDLEGRTFLHNYDWRKDKDGTLLNTIISGPALVAQWINLQYYASTVAPHFYGSGNKATQTVTSGVGVMQGNASDLMYGLSWQSVMAADRTMYHSPIRLLVVIQAPDYVVARLLANNEHFARKVSNHWLRLMSVNEEGRFKSWI | 901 | AF-Q2G0W1  (AlphaFold) | Main CO₂ uptake unit with carbonic anhydrase-like activity, essential for bicarbonate-dependent biosynthesis and pH regulation [1] |
| 2. | DabB (MpsB) | *Staphylococcus aureus* strain NCTC 8325 | MLSFQLLFSLFVIALIIALISGLLFLAPVMPMRYIKLHLYILVMPVLFAVIGFFGIHGQHVLGPFKIDRLSWLLAGFVMALGFIIQKFSMRYLLGDHHYRHYFPLFTAITSFASLAWMSEDLRLMALCWGITLLCLTLLMNVNRFWKVPRESAKLSSMTFLCGWLAFVGAIVTIYIATGEWRVPQHIVHPTWSLLTNVLLVLAVMIPAAQFPFHRWLIESVTAPTPVSAIMHAGIVNAGGVILTRFAPIFDNGFALSLLLILSSISVLLGSGISLVQVDYKRQLVGSTMSQMGFMLVQCALGVYSAAIIHLILHGIFKATLFLQSGSIVKRFNIPKQASAKDAYGWIVMGRVLAIIVAFLFWMSSDRSAYEVLSALILGWSLLVSWNQMVAFSKGRMARLVGMILIAIVTFIYVITHNYFYAVLQNITTHATTPPTVSVIISVVILIFGSLLSIWVARHRYSKAFAVLYVWLVNLGEARSKAIESHPNYLKKYL | 494 | AF-Q2G0W2  (AlphaFold) | Regulatory subunit for MpsA; possibly stabilizes or activates MpsA complex [1] |
| 3. | Cytochrome c oxidase subunit 1 | [*Thermus thermophilus HB8*](https://www.rcsb.org/search?q=rcsb_entity_source_organism.taxonomy_lineage.name:Thermus%20thermophilus%20HB8) | MHHHHHHHAVRASEISRVYEAYPEKKATLYFLVLGFLALIVGSLFGPFQALNYGNVDAYPLLKRLLPFVQSYYQGLTLHGVLNAIVFTQLFAQAIMVYLPARELNMRPNMGLMWLSWWMAFIGLVVAALPLLANEATVLYTFYPPLKGHWAFYLGASVFVLSTWVSIYIVLDLWRRWKAANPGKVTPLVTYMAVVFWLMWFLASLGLVLEAVLFLLPWSFGLVEGVDPLVARTLFWWTGHPIVYFWLLPAYAIIYTILPKQAGGKLVSDPMARLAFLLFLLLSTPVGFHHQFADPGIDPTWKMIHSVLTLFVAVPSLMTAFTVAASLEFAGRLRGGRGLFGWIRALPWDNPAFVAPVLGLLGFIPGGAGGIVNASFTLDYVVHNTAWVPGHFHLQVASLVTLTAMGSLYWLLPNLTGKPISDAQRRLGLAVVWLWFLGMMIMAVGLHWAGLLNVPRRAYIAQVPDAYPHAAVPMVFNVLAGIVLLVALLLFIYGLFSVLLSRERKPELAEAPLPFAEVISGPEDRRLVLAMDRIGFWFAVAAILVVLAYGPTLVQLFGHLNPVPGWRLWMVDEHKAHKAILAYEKGWLAFSLAMLFVFIALIAYTLATHTAGVIPAGKLERVDPTTVRQEGPWADPAQAVVQTGPNQYTVYVLAFAFGYQPNPIEVPQGAEIVFKITSPDVIHGFHVEGTNINVEVLPGEVSTVRYTFKRPGEYRIICNQYCGLGHQNMFGTIVVKEMEEKPKGALAVILVLTLTILVFWLGVYAVFFARG | 569 | 3S8F | Cytochrome c oxidase subunit 1 is the core catalytic enzyme that reduces oxygen to water and drives proton pumping for ATP generation during aerobic respiration [2] |
| 4. | Phospho-N-acetylmuramoyl-pentapeptide-transferase (MraY) | *Enterocloster bolteae* 90A9 | MGTSRDHMVLHEYVNAAGITGGSGGSENLYFQSMIHETILAIIIAFAISALLCPIIIPFLHKLKFGQQVRDDGPESHLKKQGTPTMGGLIILSSIIITSVFYIPSYPKIIPVLFVTVGFGIIGFLDDYIKIVMKRSEGLKPMQKLVGQFIITGIFAWYLLNSGEVGTDMLIPFTGGFDGGSFLSLGIFFVPALFFIMLGTDNGVNFTDGLDGLCTSVTILVATFLTIVAIGEDMGISPITGAVVGSLLGFLLFNVYPAKVFMGDTGSLALGGFVAASCYMMRMPLFIPVIGLIYLVEVLSVIIQVTYFKRTGGKRIFKMAPIHHHFELCGWSETRVVAVFAIVTAILCMVAYLGLGSGSENLYFQSHHHHHHHHHH | 376 | 5JNQ | Initiates peptidoglycan biosynthesis by transferring phospho-MurNAc-pentapeptide to undecaprenyl phosphate [3] |
| 5. | Peptide Deformylase | [*Staphylococcus aureus*](https://www.rcsb.org/search?q=rcsb_entity_source_organism.taxonomy_lineage.name:Staphylococcus%20aureus) | MLTMKDIIRDGHPTLRQKAAELELPLTKEEKETLIAMREFLVNSQDEEIAKRYGLRSGVGLAAPQINISKRMIAVLIPDDGSGKSYDYMLVNPKIVSHSVQEAYLPTGEGCLSVDDNVAGLVHRHNKITIKAKDIEGNDIQLRLKGYPAIVFQHEIDHLNGVMFYDHIDKDHPLQPHTDAVEVH | 184 | 1LMH | Removes N-formyl group from N-terminal methionine in nascent polypeptides—essential step in bacterial protein maturation [4] |
| 6. | Thymidilate Kinase (TMK) | [*Staphylococcus aureus*](https://www.rcsb.org/search?q=rcsb_entity_source_organism.taxonomy_lineage.name:Staphylococcus%20aureus) | MSAFITFEGPEGSGKTTVINEVYHRLVKDYDVIMTREPGGVPTGEEIRKIVLEGNDMDIRTEAMLFAASRREHLVLKVIPALKEGKVVLCDRYIDSSLAYQGYARGIGVEEVRALNEFAINGLYPDLTIYLNVSAEVGRERIIKNSRDQNRLDQEDLKFHEKVIEGYQEIIHNESQRFKSVNADQPLENVVEDTYQTIIKYLEKI | 205 | 4QGH | Catalyzes phosphorylation of dTMP to dTDP, crucial for DNA synthesis and repair [5] |
| 7. | SaDihydrofolate Reductase (DHFR) | [*Staphylococcus aureus*](https://www.rcsb.org/search?q=rcsb_entity_source_organism.taxonomy_lineage.name:Staphylococcus%20aureus) | TLSILVAHDLQRVIGFENQLPWHLPNDLKHVKKLSTGHTLVMGRKTFESIGKPLPNRRNVVLTSDTSFNVEGVDVIHSIEDIYQLPGHVFIFGGQTLYEEMIDKVDDMYITVIEGKFRGDTFFPPYTFEDWEVASSVEGKLDEKNTIPHTFLHLIRK | 157 | 3F0U | Reduces dihydrofolate to tetrahydrofolate; critical for thymidine and purine synthesis [6] |
| 8. | Lipoprotein Signal Peptidase-II (LspA) | [*Staphylococcus aureus*](https://www.rcsb.org/search?q=rcsb_entity_source_organism.taxonomy_lineage.name:Staphylococcus%20aureus) | MGHHHHHHDYDIPTTENLYFQGAHMHKKYFIGTSILIAVFVVIFDQVTKYIIATTMKIGDSFEVIPHFLNITSHRNNGAAWGILSGKMTFFFIITIIILIALVYFFIKDAQYNLFMQVAISLLFAGALGNFIDRVLTGEVVDFIDTNIFGYDFPIFNIADSSLTIGVILIIIALLKDTSNKKEKEVK | 187 | 6RYO | Cleaves signal peptides from prolipoproteins; vital for lipoprotein maturation and membrane insertion [7] |
| 9. | DNA Topoisomerase IV, B Subunit | [*Staphylococcus aureus*](https://www.rcsb.org/search?q=rcsb_entity_source_organism.taxonomy_lineage.name:Staphylococcus%20aureus) | MAMNKQNNYSDDSIQVLEGLEAVRKRPGMYIGSTDKRGLHHLVYEIVDNSVDEVLNGYGNEIDVTINKDGSISIEDNGRGMPTGIHKSGKPTVEVIFTVLHAGGKFGQGGYKTSGGLHGVGASVVNALSEWLEVEIHRDGNIYHQSFKNGGSPSSGLVKKGKTKKTGTKVTFKPDDTIFKASTSFNFDVLSERLQESAFLLKNLKITLNDLRSGKERQEHYHYEE | 225 | 4URN | Relieves supercoiling during DNA replication and chromosome segregation [8] |
| 10. | DNA Gyrase Subunit B  (GyrB) | [*Staphylococcus aureus*](https://www.rcsb.org/search?q=rcsb_entity_source_organism.taxonomy_lineage.name:Staphylococcus%20aureus) | MVTALSDVNNTDNYGAGQIQVLEGLEAARKRPGMYIGSTSERGLHHLVWEIVDNSIDEALAGYANQIEVVIEKDNWIKVTDNGRGIPVDIQEKMGRPAVEVILTVLHAGGKFGGGGYKVSGGLHGVGSSVVNALSQDLEVYVHRNETIYHQAYKKGVPQFDLKEVGTTDKTGTVIRFKADGEIFTETTVYNYETLQQRIRELAFLNKGIQITLRDERDEENVREDSYHYEG | 231 | 4URO | DNA supercoiling and negative winding; works with GyrA in DNA replication [8] |
| 11. | Cell division protein FtsZ | [*Staphylococcus aureus*](https://www.rcsb.org/search?q=rcsb_entity_source_organism.taxonomy_lineage.name:Staphylococcus%20aureus) | MHHHHHHLEFEQGFNHLATLKVIGVGGGGNNAVNRMIDHGMNNVEFIAINTDGQALNLSKAESKIQIGEKLTRGLGAGANPEIGKKAAEESREQIEDAIQGADMVFVTSGMGGGTGTGAAPVVAKIAKEMGALTVGVVTRPFSFEGRKRQTQAAAGVEAMKAAVDTLIVIPNDRLLDIVDKSTPMMEAFKEADNVLRQGVQGISDLIAVSGEVNLDFADVKTIMSNQGSALMGIGVSSGENRAVEAAKKAISSPLLETSIVGAQGVLMNITGGESLSLFEAQEAADIVQDAADEDVNMIFGTVINPELQDEIVVTVIATGFDDKPTSHGRKSGSTGFGTSVNTSSNATSKDESFTSNSSNAQATDSVSERTHTTKEDDIPSFIRNREERRSRRTRR | 396 | 4DXD | Tubulin homolog forming the Z-ring—essential scaffold for bacterial cytokinesis [9] |
| 12. | Outer Membrane Protein Assembly Factor BamA | *Escherichia coli* | HMRNTGSFNFGIGYGTESGVSFQAGVQQDNWLGTGYAVGINGTKNDYQTYAELSVTNPYFTVDGVSLGGRLFYNDFQADDADLSDYTNKSYGTDVTLGFPINEYNSLRAGLGYVHNSLSNMQPQVAMWRYLYSMGEHPSTSDQDNSFKTDDFTFNYGWTYNKLDRGYFPTDGSRVNLTGKVTIPGSDNEYYKVTLDTATYVPIDDDHKWVVLGRTRWGYGDGLGGKEMPFYENFYAGGSSTVRGFQSNTIGPKAVYFPHQASNYDPDYDYESATQDGAKDLSKSDDAVGGNAMAVASLEFITPTPFISDKYANSVRTSFFWDMGTVWDTNWDSSQYSGYPDYSDPSNIRMSAGIALQWMSPLGPLVFSYAQPFKKYDGDKAEQFQFNIGKTW | 392 | 7R1V | Catalyzes folding and insertion of outer membrane proteins (OMPs) in Gram-negative bacteria [10] |
| 13. | MurE | *Escherichia coli* K-12 | SMADRNLRDLLAPWVPDAPSRALREMTLDSRVAAAGDLFVAVVGHQADGRRYIPQAIAQGVAAIIAEAKDEATDGEIREMHGVPVIYLSQLNERLSALAGRFYHEPSDNLRLVGVTGTNGKTTTTQLLAQWSQLLGEISAVMGTVGNGLLGKVIPTENTTGSAVDVQHELAGLVDQGATFCAMEVSSHGLVQHRVAALKFAASVFTNLSRDHLDYHGDMEHYEAAKWLLYSEHHCGQAIINADDEVGRRWLAKLPDAVAVSMEDHINPNCHGRWLKATEVNYHDSGATIRFSSSWGDGEIESHLMGAFNVSNLLLALATLLALGYPLADLLKTAARLQPVCGRMEVFTAPGKPTVVVDYAHTPDALEKALQAARLHCAGKLWCVFGCGGDRDKGKRPLMGAIAEEFADVAVVTDDNPRTEEPRAIINDILAGMLDAGHAKVMEGRAEAVTCAVMQAKENDVVLVAGKGHEDYQIVGNQRLDYSDRVTVARLLGVIA | 496 | 7B6M | Adds meso-diaminopimelate or lysine to peptidoglycan precursor; part of cell wall synthesis  (To be published) |
| 14. | Macrolide 2'-phosphotransferase I | *Escherichia coli* | MTVVTTADTSQLYALAARHGLKLHGPLTVNELGLDYRIVIATVDDGRRWVLRIPRRAEVSAKVEPEARVLAMLKNRLPFAVPDWRVANAELVAYPMLEDSTAMVIQPGSSTPDWVVPQDSEVFAESFATALAALHAVPISAAVDAGMLIRTPTQARQKVADDVDRVRREFVVNDKRLHRWQRWLDDDSSWPDFSVVVHGDLYVGHVLIDNTERVSGMIDWSEARVDDPAIDMAAHLMVFGEEGLAKLLLTYEAAGGRVWPRLAHHIAERLAFGAVTYALFALDSGNEEYLAAAKAQLAAAE | 301 | 5IGH | Inactivates macrolides via phosphorylation—antibiotic resistance mechanism [11] |
| 15. | LpxC | *Pseudomonas aeruginosa* [PAO1](https://www.rcsb.org/search?q=rcsb_entity_source_organism.taxonomy_lineage.name:Pseudomonas%20aeruginosa%20PAO1) | MIKQRTLKNIIRATGVGLHSGEKVYLTLKPAPVDTGIVFSRTDLDPVVEIPARAENVGETTMSTTLVKGDVKVDTVEHLLSAMAGLGIDNAYVELSASEVPIMDGSAGPFVFLIQSAGLQEQEAAKKFIRIKREVSVEEGDKRAVFVPFDGFKVSFEIDFDHPVFRGRTQQASVDFSSTSFVKEVSRARTFGFMRDIEYLRSQNLALGGSVENAIVVDENRVLNEDGLRYEDEFVKHKILDAIGDLYLLGNSLIGEFRGFKSGHALNNQLLRTLIADKDAWEVVTFEDARTAPISYMRP | 299 | 2VES | Zinc metalloenzyme; catalyzes lipid A biosynthesis in Gram-negative bacteria—virulence determinant [12] |
| 16. | β-Carbonic anhydrase | [*Vibrio cholerae*](https://www.rcsb.org/search?q=rcsb_entity_source_organism.taxonomy_lineage.name:Vibrio%20cholerae) | MPEIKQLFENNSKWSASIKAETPEYFAKLAKGQNPDFLWIGCADSRVPAERLTGLYSGELFVHRNVANQVIHTDLNCLSVVQYAVDVLQVKHIIVCGHYGCGGVTAAIDNPQLGLINNWLLHIRDYYLKHREYLDKMPAEDRSDKLAEINVAEQVYNLANSTVLQNAWERGQAVEVHGFVYGIEDGRLEYLGVRCASRSAVEDNYHKALEKILNPNHRLLCR | 222 | 5CXK | Reversibly converts CO₂ to HCO₃⁻; contributes to pH regulation and CO₂ transport [13] |
| 17. | Sterol 14-α-Demethylase (CYP51) | *Candida albicans* | MAKKTPPLVFYWIPWFGSAASYGQQPYEFFESCRQKYGDVFSFMLLGKIMTVYLGPKGHEFVFNAKLSDVSAEEAYKHLTTPVFGTGVIYDCPNSRLMEQKKFAKFALTTDSFKRYVPKIREEILNYFVTDESFKLKEKTHGVANVMKTQPEITIFTASRSLFGDEMRRIFDRSFAQLYSDLDKGFTPINFVFPNLPLPHYWRRDAAQKKISATYMKEIKLRRERGDIDPNRDLIDSLLIHSTYKDGVKMTDQEIANLLIGILMGGQHTSASTSAWFLLHLGEKPHLQDVIYQEVVELLKEKGGDLNDLTYEDLQKLPSVNNTIKETLRMHMPLHSIFRKVTNPLRIPETNYIVPKGHYVLVSPGYAHTSERYFDNPEDFDPTRWDTAAAKANSVSFNSSDEVDYGFGKVSKGVSSPYLPFGGGRHRCIGEQFAYVQLGTILTTFVYNLRWTIDGYKVPDPDYSSMVVLPTEPAEIIWEKRETCMFHHHH | 490 | 5TZ1 | Key enzyme in ergosterol biosynthesis—fungal membrane stability target [14] |
| 18. | Secreted Aspartic Proteinase | *Candida albicans* | QAVPVTLHNEQVTYAADITVGSNNQKLNVIVDTGSSDLWVPDVNIDCQVTYSDQTADFCKQKGTYDPSGSSASQDLNTPFSIGYGDGSSSQGTLYKDTVGFGGVSIKNQVLADVDSTSIDQGILGVGYKTNEAGGSYDNVPVTLKKQGVIAKNAYSLYLNSPDSATGQIIFGGVDNAKYSGSLIALPVTSDRELRISLGSVEVSGKTINTDNVDVLLDSGTTITYLQQDLADQIIKAFNGKLTQDSNGNSFYEVDCNLSGDVVFNFSKNAKISVPASDFAASTQGDDGQPYDKCQLLFDVNKANILGDNFLRSAYIVYDLDDNEISIAQVKYTSASSTSALT | 342 | 1ZAP | Hydrolyzes host proteins; contributes to fungal invasion and immune evasion [15] |
| 19. | *C. albicans* Dihydrofolate Reductase (CaDHFR) | *Candida albicans* | MLKPNVAIIVAALKPALGIGYKGKMPWRLRKEIRYFKDVTTRTTKPNTRNAVIMGRKTWESIPQKFRPLPDRLNIILSRSYENEIIDDNIIHASSIESSLNLVSDVERVFIIGGAEIYNELINNSLVSHLLITEIEHPSPESIEMDTFLKFPLESWTKQPKSELQKFVGDTVLEDDIKEGDFTYNYTLWTRK | 192 | 3QLS | Same as bacterial DHFR; essential for folate metabolism and DNA synthesis in fungi [16] |
| 20. | Serine/Threonine Phosphatase Z1 (PPZ1) | *Candida albicans* | GHMIDIDSLIDKLLNAGFSGKRTKNVCLKNTEIELICASAREIFLSQPSLLELAPPVKVVGDVHGQYHDLIRIFSKCGFPPKTNYLFLGDYVNRGKQSLETILLLLCYKIKYPENFFLLRGNHECANVTRVYGFYDECKRRCNIKTWKLFIDTFNTLPIAAIVAGKIFCVHGGLSPVLNSMDEIRNIARPTDVPDFGLLNDLLWSDPADTINEWEDNERGVSYVFSKVAINKFLSKFNFDLVCRAHMVVEDGYEFFNDRTLVTVFSAPNYCGEFDNWGAVMGVSEDLLCSFELLDPLDSAALKQVMKKEKQERKKST | 317 | 5JPF | Regulates ion homeostasis, cell wall integrity, and stress response in fungi; protects against oxidative stress [17,18] |
| 21. | Exo-β-(1,3)-Glucanase | *Candida albicans* | AWDYDNNVIRGVNLGGWFVLEPYMTPSLFEPFQNGNDQSGVPVDEYHWTQTLGKEAASRILQKHWSTWITEQDFKQISNLGLNFVRIPIGYWAFQLLDNDPYVQGQVQYLEKALGWARKNNIRVWIDLHGAPGSQNGFDNSGLRDSYNFQNGDNTQVTLNVLNTIFKKYGGNEYSDVVIGIELLNEPLGPVLNMDKLKQFFLDGYNSLRQTGSVTPVIIHDAFQVFGYWNNFLTVAEGQWNVVVDHHHYQVFSGGELSRNINDHISVACNWGWDAKKESHWNVAGEWSAALTDCAKWLNGVNRGARYEGAYDNAPYIGSCQPLLDISQWSDEHKTDTRRYIEAQLDAFEYTGGWVFWSWKTENAPEWSFQTLTYNGLFPQPVTDRQFPNQCGFH | 394 | 1EQP | Involved in fungal cell wall remodeling during hyphal growth and conidial germination [19,20] |
| 22. | N-Myristoyl Transferase (NMT) | *Aspergillus fumigatus* | GPRSQTQPVPRFDETSTDTGGPIKIIDPEKVSKEPDALLEGFEWATLDLTNETELQELWDLLTYHYVEDDNAMFRFRYSQSFLHWALMSPGWKKEWHVGVRATKSRKLVASICGVPTEINVRNQKLKVVEINFLCIHKKLRSKRLTPVLIKEITRRCYLNGIYQAIYTAGVVLPTPVSSCRYYHRPLDWLKLYEVGFSPLPAGSTKARQITKNHLPSTTSTPGLRPMEPKDIDTVHDLLQRYLSRFALNQAFTREEVDHWLVHKPETVKEQVVWAYVVEDPETHKITDFFSFYNLESTVIQNPKHDNVRAAYLYYYATETAFTNNMKALKERLLMLMNDALILAKKAHFDVFNALTLHDNPLFLEQLKFGAGDGQLHFYLYNYRTAPVPGGVNEKNLPDEKRMGGVGIVML | 411 | 4CAV | Transfers myristoyl group to proteins; critical for protein localization and cell wall biosynthesis [21] |
| 23. | *A. flavus* Squalene Synthase (AfSQS) | *Aspergillus flavus* | MRATEVLYYMLRPSQLRSIVQWKVWHNPVHERNVNNETETQKACFKFLDLTSRSFSAVIKELHPELLLPVCVFYLVLRGLDTIEDDTSIPLKTKEPMLREFKDYLEQDGWTFDGNRPEEKDRELLVQFHNVITEFKNMKPAYREIVKDITDKMGNGMADYCRKAEFEDASVKTIEEYDLYCYYVAGLVGEGLTRLFVEAEFGNPALLSRPRLHKSMGLFLQKTNIIRDVREDHDDDRHFWPKEIWSKYVTEFEDLFKPENRETALNCGSEMVLNALEHAEECLFYLAGLREQSVFNFCAIPQAMAIATLELCFRNPDMFDRNIKITKGEACQLMMESTQNLHVLCDTFRRYARRIHKKNTPKDPNFLKISIVCGKIEKFIDTIFPQQTAAQAKLKVQGEKSEAEKEKARQEAETRQDLYFMLALMGVIVLIVSIIMLTAAWLLGARFDLAFQELKSGNFRPPAKQIPGEL | 470 | 7WGH | Catalyzes the first step in ergosterol biosynthesis, a key antifungal target [22] |

**References**

1. Fan S-H, Ebner P, Reichert S, Hertlein T, Zabel S, Lankapalli AK, et al. MpsAB is important for Staphylococcus aureus virulence and growth at atmospheric CO2 levels. Nat Commun. 2019;10: 3627. doi:10.1038/s41467-019-11547-5

2. Shimada A, Tsukihara T, Yoshikawa S. Copper in Biology: Molecular Structures, Cellular Processes and Living Systems. Reaction Mechanism of Cytochrome c Oxidase. Royal Society of Chemistry; 2025. p. 356. Available: https://books.rsc.org/books/edited-volume/2327/chapter/8581700/Reaction-Mechanism-of-Cytochrome-c-Oxidase

3. Hakulinen JK, Hering J, Brändén G, Chen H, Snijder A, Ek M, et al. MraY–antibiotic complex reveals details of tunicamycin mode of action. Nat Chem Biol. 2017;13: 265–267. doi:10.1038/nchembio.2270

4. Baldwin ET, Harris MS, Yem AW, Wolfe CL, Vosters AF, Curry KA, et al. Crystal structure of type II peptide deformylase from Staphylococcus aureus. Journal of Biological Chemistry. 2002;277: 31163–31171. doi:10.1074/jbc.M202750200

5. Antibacterial Inhibitors of Gram-Positive Thymidylate Kinase: Structure–Activity Relationships and Chiral Preference of a New Hydrophobic Binding Region | Journal of Medicinal Chemistry. [cited 20 Oct 2024]. Available: https://pubs.acs.org/doi/10.1021/jm500463c

6. Frey KM, Liu J, Lombardo MN, Bolstad DB, Wright DL, Anderson AC. Crystal Structures of Wild-type and Mutant Methicillin-resistant Staphylococcus aureus Dihydrofolate Reductase Reveal an Alternate Conformation of NADPH That May Be Linked to Trimethoprim Resistance. Journal of Molecular Biology. 2009;387: 1298–1308. doi:10.1016/j.jmb.2009.02.045

7. Olatunji S, Yu X, Bailey J, Huang C-Y, Zapotoczna M, Bowen K, et al. Structures of lipoprotein signal peptidase II from Staphylococcus aureus complexed with antibiotics globomycin and myxovirescin. Nat Commun. 2020;11: 140. doi:10.1038/s41467-019-13724-y

8. Lu J, Patel S, Sharma N, Soisson SM, Kishii R, Takei M, et al. Structures of kibdelomycin bound to Staphylococcus aureus GyrB and ParE showed a novel U-shaped binding mode. ACS Chemical Biology. 2014;9: 2023–2031. doi:10.1021/cb5001197

9. Tan CM, Therien AG, Lu J, Lee SH, Caron A, Gill CJ, et al. Restoring Methicillin-Resistant Staphylococcus aureus Susceptibility to β-Lactam Antibiotics. Science Translational Medicine. 2012;4: 126ra35-126ra35. doi:10.1126/scitranslmed.3003592

10. Miller RD, Iinishi A, Modaresi SM, Yoo BK, Curtis TD, Lariviere PJ, et al. Computational identification of a systemic antibiotic for gram-negative bacteria. Nature Microbiology. 2022;7: 1661–1672. doi:10.1038/s41564-022-01227-4

11. Fong DH, Burk DL, Blanchet J, Yan AY, Berghuis AM. Structural Basis for Kinase-Mediated Macrolide Antibiotic Resistance. Structure. 2017;25: 750-761.e5. doi:10.1016/j.str.2017.03.007

12. Mochalkin I, Knafels JD, Lightle S. Crystal structure of LpxC from Pseudomonas aeruginosa complexed with the potent BB-78485 inhibitor. Protein Science. 2008;17: 450–457. doi:10.1110/ps.073324108

13. Ferraroni M, Del Prete S, Vullo D, Capasso C, Supuran CT. Crystal structure and kinetic studies of a tetrameric type II β-carbonic anhydrase from the pathogenic bacterium Vibrio cholerae. Acta Crystallographica Section D: Biological Crystallography. 2015;71: 2449–2456. doi:10.1107/S1399004715018635

14. Hargrove TY, Friggeri L, Wawrzak Z, Qi A, Hoekstra WJ, Schotzinger RJ, et al. Structural analyses of Candida albicans sterol 14α-demethylase complexed with azole drugs address the molecular basis of azole-mediated inhibition of fungal sterol biosynthesis. Journal of Biological Chemistry. 2017;292: 6728–6743. doi:10.1074/jbc.M117.778308

15. Abad-Zapatero C, Goldman R, Muchmore SW, Hutchins C, Stewart K, Navaza J, et al. Structure of a secreted aspartic protease from C. albicans complexed with a potent inhibitor: Implications for the design of antifungal agents. Protein Science. 1996;5: 640–652. doi:10.1002/pro.5560050408

16. Paulsen JL, Bendel SD, Anderson AC. Crystal Structures of Candida albicans Dihydrofolate Reductase Bound to Propargyl-Linked Antifolates Reveal the Flexibility of Active Site Loop Residues Critical for Ligand Potency and Selectivity. Chemical Biology and Drug Design. 2011;78: 505–512. doi:10.1111/j.1747-0285.2011.01169.x

17. Chen E, Choy MS, Petrényi K, Kónya Z, Erdődi F, Dombrádi V, et al. Molecular insights into the fungus-specific serine/threonine protein phosphatase Z1 in Candida albicans. mBio. 2016;7. doi:10.1128/mBio.00872-16

18. Hajdu T, Szabó K, Jakab Á, Pócsi I, Dombrádi V, Nagy P. Biophysical experiments reveal a protective role of protein phosphatase Z1 against oxidative damage of the cell membrane in Candida albicans. Free Radical Biology and Medicine. 2021;176: 222–227. doi:10.1016/j.freeradbiomed.2021.09.020

19. Cutfield JF, Sullivan PA, Cutfield SM. Minor structural consequences of alternative CUG codon usage (Ser for Leu) in Candida albicans exoglucanase. Protein Engineering. 2000;13: 735–738. doi:10.1093/protein/13.10.735

20. Mouyna I, Hartl L, Latgé JP. β-1,3-glucan modifying enzymes in Aspergillus fumigatus. Frontiers in Microbiology. Frontiers Research Foundation; 2013. p. 81. doi:10.3389/fmicb.2013.00081

21. Fang W, Robinson DA, Raimi OG, Blair DE, Harrison JR, Lockhart DEA, et al. N -Myristoyltransferase Is a Cell Wall Target in Aspergillus fumigatus. ACS Chemical Biology. 2015;10: 1425–1434. doi:10.1021/cb5008647

22. Malwal SR, Shang N, Liu W, Li X, Zhang L, Chen CC, et al. A Structural and Bioinformatics Investigation of a Fungal Squalene Synthase and Comparisons with Other Membrane Proteins. ACS Omega. 2022;7: 22601–22612. doi:10.1021/acsomega.2c01924
