# Supplementary material for: Antimicrobial and antibiofilm evaluation of thymol, sodium azide, and sodium lauryl sulfate against multidrug-resistant pathogens: An integrated experimental and computational study
Source: PLoS One. 2026 Apr 7;21(4):e0345977. doi: 10.1371/journal.pone.0345977 (PMC13056186; doi:10.1371/journal.pone.0345977)
Supplement: S2 Table — This table summarizes the structural validation parameters for the AlphaFold-predicted models of DabA and DabB, including the total number of residues, Rg, mean and median pLDDT scores, and the proportion of residues within high-confidence (≥90), confident (70–89), and low-confidence (<50) ranges. Both proteins exhibit compact folds and high overall prediction confidence, with mean pLDDT scores of 87.92 for DabA and 88.69 for DabB and minimal low-confidence regions. These metrics support the suitability of the models for downstream molecular docking and molecular dynamics simulations. (DOCX) [file pone.0345977.s033.docx]

| **#** | **Model** | **Residues with Cα** | **Radius of gyration (Å)** | **Mean pLDDT** | **Median pLDDT** | **Fraction pLDDT (≥90)** | **Fraction pLDDT (≥70)** | **Fraction pLDDT (<50)** |
| --- | --- | --- | --- | --- | --- | --- | --- | --- |
| **1** | **AF-Q2G0W1-F1-model_v6_DabA(MpsA)** | 901 | 31.709 | 87.92 | 93.31 | 0.666 | 0.921 | 0.057 |
| **2** | **AF-Q2G0W2-F1-model_v6_DabB(MpsB)** | 494 | 23.229 | 88.69 | 91.38 | 0.573 | 0.957 | 0.000 |
