# Supplementary material for: Antimicrobial and antibiofilm evaluation of thymol, sodium azide, and sodium lauryl sulfate against multidrug-resistant pathogens: An integrated experimental and computational study
Source: PLoS One. 2026 Apr 7;21(4):e0345977. doi: 10.1371/journal.pone.0345977 (PMC13056186; doi:10.1371/journal.pone.0345977)
Supplement: Attachment — Submitted filename: Response to Reviewers.pdf [file pone.0345977.s036.pdf]

Response to Reviewer's Comments

We sincerely thank the esteemed reviewers for taking the time to evaluate our manuscript. We truly appreciate their dedication, thoughtful assessments, and constructive insights. The comments have greatly strengthened the quality, clarity, and overall presentation of the work.

We have carefully addressed each point and revised the manuscript accordingly. Our detailed responses to the reviewers' comments are provided below:

Journal Requirements

Comment	Response
When submitting your revision, we need you to address these additional requirements.<br><br>1.Please ensure that your manuscript meets PLOS ONE's style requirements, including those for file naming. The PLOS ONE style templates can be found at<br><br>https://journals.plos.org/plosone/s/file?id=wjVg/PLOSOne_formatting_sample_main_body.pdf and<br><br>https://journals.plos.org/plosone/s/file?id=ba62/PLOSOne_formatting_sample_title_authors_affiliations.pdf	We appreciate the reminder regarding PLOS ONE's formatting and file-naming requirements. We have carefully revised the manuscript and ensured that all files now fully comply with the journal's style guidelines and templates.

2. Thank you for stating the following financial disclosure:<br><br>"The Researchers would like to thank the Deanship of Graduate Studies and Scientific Research at Qassim University for their financial support (QU-APC-2025)."<br><br>Please state what role the funders took in the study. If the funders had no role, please state: "The funders had no role in study design, data collection and analysis, decision to publish, or preparation of the manuscript."<br><br>If this statement is not correct you must amend it as needed.<br><br>Please include this amended Role of Funder statement in your cover letter; we will change the online submission form on your behalf.	Thank you for the clarification. We confirm that:<br><br>**The funders had no role in study design, data collection and analysis, decision to publish, or preparation of the manuscript.**<br><br>We have included this amended Role of Funder statement in the cover letter as requested.<br><br>The corrected financial disclosure statement is :<br><br>**The Researchers would like to thank the Deanship of Graduate Studies and Scientific Research at Qassim University for financial support (QU-APC-2025).**

3. We note that your Data Availability Statement is currently as follows: All relevant data are within the manuscript and in Supporting Information files.<br><br>Please confirm at this time whether or not your submission contains all raw data required to replicate the results of your study. Authors must share the “minimal data set” for their submission. PLOS defines the minimal data set to consist of the data required to replicate all study findings reported in the article, as well as related metadata and methods (https://journals.plos.org/plosone/s/data-availability#loc-minimal-data-set-definition).<br><br>For example, authors should submit the following data:<br><br>- The values behind the means, standard deviations and other measures reported;<br><br>- The values used to build graphs;<br><br>- The points extracted from images for analysis.<br><br>Authors do not need to submit their entire data set if only a portion of the data was used in the reported study.	We confirm that all data required to replicate the findings of this study have been provided in the main manuscript and in the Supporting Information files. These include all numerical values underlying the reported means, standard deviations, graphs, and analyses.<br><br>The amended Data Availability Statement is as follows:<br><br>**All relevant data are included within the manuscript and its Supporting Information files. These files contain all numerical values and datasets required to reproduce the study’s results. Additional raw data—such as full molecular dynamics simulation trajectories and associated large-scale files—are not included due to their substantial size but can be provided to the editor, reviewers, or interested researchers upon reasonable request.**

If your submission does not contain these data, please either upload them as Supporting Information files or deposit them to a stable, public repository and provide us with the relevant URLs, DOIs, or accession numbers. For a list of recommended repositories, please see https://journals.plos.org/plosone/s/recommended-repositories.<br><br>If there are ethical or legal restrictions on sharing a de-identified data set, please explain them in detail (e.g., data contain potentially sensitive information, data are owned by a third-party organization, etc.) and who has imposed them (e.g., an ethics committee). Please also provide contact information for a data access committee, ethics committee, or other institutional body to which data requests may be sent. If data are owned by a third party, please indicate how others may request data access.	All essential data required to replicate the study have been included in the manuscript and the Supporting Information files. Additional raw data, such as the full molecular dynamics simulation trajectories, are extremely large and therefore not uploaded to the repository at this stage; however, these files can be provided to the editor or reviewers upon request. There are no ethical or legal restrictions on sharing the data.
4. If the reviewer comments include a recommendation to cite specific previously published works, please review and evaluate these publications to determine whether they are relevant and should be cited. There is no requirement to cite these works unless the editor has indicated otherwise.	Regarding the reviewer-suggested citations, we have carefully evaluated the recommended publications and have cited them where appropriate.

Reviewer-1

Scientific terms such as in vitro, in vivo, and in silico should be italicized throughout the manuscript. Please check and revise accordingly.	Thank you for the suggestion. We have carefully revised the manuscript and ensured that all scientific terms, including *in vitro*, *in vivo*, and *in silico*, are italicized throughout.
The images currently provided have colored backgrounds. It would be preferable to present them with a plain (white or transparent) background for better clarity and uniformity.	We have updated all images to have a plain white/transparent background to ensure clarity and uniformity.
Please verify whether AlphaFold structures are available for the MpsA and MpsB proteins. If available, use these structures for the computational analyses.	Yes, AlphaFold has released predicted structures for both **MpsA** and **MpsB**. These proteins are listed in AlphaFold under their updated names: **DabA** (formerly MpsA) and **DabB** (formerly MpsB). We have now incorporated these AlphaFold models into our computational workflow. Accordingly, we have adopted the updated nomenclature in the manuscript and used the AlphaFold structures of **DabA (MpsA)** and **DabB (MpsB)**, the two subunits of the bicarbonate transporter system.
A detailed molecular dynamics (MD) simulation protocol should be provided, including the parameters used for energy minimization, equilibration, and production phases.	We appreciate the reviewer's suggestion. A complete and detailed MD simulation protocol has now been added to the Methods section.
Clearly specify whether the MM-GBSA or MM-PBSA method was used for binding free energy calculations.	We confirm that **MM-GBSA** was used for all binding free energy calculations, and this has already been clearly stated in the Methods section.
Table 1 should be expanded to include hydrogen-bonding residues along with their bond distances. Additionally, check for possible π–π stacking interactions and include them in the table if present.	Table 1 has been revised to include, for each docked complex, the hydrogen-bonding residues with their bond distances, π–π stacking residues with distances, as well as the residues involved

	in hydrophilic and hydrophobic interactions, salt bridges, and water-mediated hydrogen bonds.
Certain parts of the Results section are presented as bullet points. Please rewrite these sections in paragraph form for improved readability.	Thank you for this helpful comment. All bullet-pointed text within the Results section has now been rewritten into complete paragraphs to improve readability and maintain consistency.
Some words in the Results section are highlighted in bold. Please remove the bold formatting.	Resolved.
In Lines 388–390, you state:<br><br>"RMSF analyses (Figs. 17–19B) showed reduced fluctuations in active-site residues for 4URN–PA and 5TZ1–PA, highlighting the ability of PA to stabilize key residues, particularly those involved in hydrogen bonding and hydrophobic interactions."<br><br>Please visually highlight the reduced fluctuations of active-site residues in the RMSF plot for clarity.	We thank the reviewer for this helpful suggestion. To address the request without altering the published RMSF figures, we have now explicitly identified and described the stabilized active-site and pocket residues within the Results section. Specifically, the revised text now highlights that 4URN–PA exhibits reduced fluctuations in ASP76, GLY80, and MET81, while 5TZ1–PA shows reduced flexibility in LEU376, ILE379, ARG381, PHE463, GLY464, HIS468, and CYS470. These residues correspond precisely to the binding-site region indicated in Table 1.<br>By directly naming the stabilized residues and relating them to the RMSF profiles in Figs. 17–19B, the revised manuscript provides clear interpretive guidance for readers while preserving the integrity of the original figures. This revision resolves the reviewer's concern and enhances the clarity of the RMSF analysis.
The Results and Discussion section on MD simulations lacks sufficient statistical detail. Please include information such as, Equilibration time and pattern observed, Residues showing higher flexibility, and Average number of hydrogen bonds maintained during the simulation.	We sincerely thank the reviewer for this valuable suggestion. In the revised manuscript, we have now incorporated detailed statistical descriptions within the MD simulation section. Specifically, we added: **(i)** the equilibration time and RMSD stabilization patterns observed across all trajectories, **(ii)** the identification of flexible regions and key residues based on RMSF profiles, and **(iii)** the

	average and ligand-specific patterns of hydrogen bonds maintained during the 300-ns simulations.
The discussion on PCA-based FEL (Free Energy Landscape) analysis is insufficient. Please provide a more detailed explanation of the FEL plots in terms of conformational stability and energy minima of the complexes.	We thank the reviewer for this insightful comment**.** In accordance with the suggestion, we have substantially expanded the PCA-based Free Energy Landscape (FEL) analysis to provide a clearer and more comprehensive interpretation of the conformational stability of all protein–ligand complexes. The revised manuscript now includes: **(i)** a detailed explanation of the principal conformational states derived from PCA projections along PC1 and PC2, **(ii)** an in-depth interpretation of the FEL plots, highlighting the location, depth, and distribution of the dominant energy minima (basins), and **(iii)** a systematic discussion of how these energy minima correlate with the stability, flexibility, and conformational preferences of each complex. This expanded analysis clarifies the relationship between essential motions and structural stabilization, thereby addressing the reviewer's concern and improving the overall mechanistic understanding of the MD simulation results.
Authors are requested to deposit their MD simulation trajectories and associated data in a publicly accessible repository (e.g., Zenodo, Figshare, or Mendeley Data) and provide the corresponding access link within the manuscript for transparency and reproducibility.	We appreciate the reviewer's valuable suggestion regarding data transparency. Due to the large size of the MD simulation trajectories and associated files, we are unable to upload them to a public repository. However, we confirm that all raw MD trajectory files, input parameters, topology files, and analysis outputs (including RMSD, RMSF, Rg, SASA, hydrogen-bond statistics, PCA and FEL results, and MM-GBSA calculations) are fully available on request by reviewers, editors or readers.

	A revised statement has now been added to the **Data Availability** section of the manuscript:<br><br>**All relevant data are included within the manuscript and its Supporting Information files. These files contain all numerical values and datasets required to reproduce the study's results. Additional raw data—such as full molecular dynamics simulation trajectories and associated large-scale files—are not included due to their substantial size but can be provided to the editor, reviewers, or interested researchers upon reasonable request.**

Reviewer-2

The manuscript states that an RMSD ≤ 2.0 Å was used as the criterion for successful re-docking validation. Could the authors elaborate on why the goal was not to achieve a 0 Å RMSD, or clarify the rationale behind choosing the 2.0 Å threshold instead?	We thank the reviewer for this query. In MOE tool, achieving a 0 Å RMSD during re-docking is not expected because the software uses stochastic search algorithms and energy-minimization steps that naturally introduce small deviations from the crystallographic pose. Therefore, an RMSD ≤ 2.0 Å is the widely accepted benchmark in MOE-based validation studies, indicating that the docking protocol reliably reproduces the native binding orientation within experimentally acceptable limits.
The authors mention that the structures of MpsA and MpsB were modeled using SWISS-MODEL. Could the authors clarify why AlphaFold, which generally provides higher-accuracy structural predictions, was not used as an alternative or for comparison?	We thank the reviewer for the insightful comment. We acknowledge that AlphaFold generally provides high-accuracy structural predictions. In our initial workflow, the SWISS-MODEL structures of MpsA and MpsB were used because, at that time, AlphaFold entries for these proteins (now renamed **DabA** and **DabB**) were not yet available in the database we checked. After receiving the reviewer's request, we verified the availability of AlphaFold models and confirmed that high-confidence structures are indeed provided for both subunits.<br><br>We have now updated our computational analyses using the AlphaFold structures of **DabA (formerly MpsA)** and **DabB (formerly MpsB)**. These models offer superior prediction quality and confidence metrics, and therefore represent a more reliable basis for downstream docking and MD simulations. The manuscript has been revised accordingly, with updated nomenclature and justification.

The manuscript currently includes a large number of figures in the main text. To improve readability and streamline the presentation, it is recommended to reduce the number of figures in the main manuscript to a maximum of 12. The remaining figures can be moved to the supplementary material.	We thank the reviewer for this helpful suggestion. To improve clarity and streamline the manuscript, we have reduced the number of figures in the main text to **13**, as recommended. All additional figures—particularly those related to extended RMSD/RMSF analyses, secondary plots, and supplementary PCA/FEL panels—have now been moved to the **Supporting Information** while ensuring that all essential results remain clearly presented in the main manuscript. The figure numbering has been updated accordingly.

Reviewer-3

The title need to be revised. There are repetitions such as in silico and computational, in vitro and the assays stated! Shorten the title to a sentence that covers the whole study.	Thank you for this valuable comment. We appreciate the reviewer's suggestion to remove redundancies and make the title more concise. In response, we have revised the title to avoid repetition of terms such as "*in vitro*," "*in silico*," and overlapping descriptions of assays. The new title provides a clear, single-sentence summary that captures the overall scope of the work.<br><br>**"Antimicrobial and Antibiofilm Evaluation of Thymol, Sodium Azide, and Sodium Lauryl Sulfate Against Multidrug-Resistant Pathogens: An Integrated Experimental and Computational Study"**
The compounds TM, SA, SLS, PA, LS should be fully abbreviated when mentioned in the first place.	Thank you for your helpful observation. We agree with the reviewer's recommendation. In the revised manuscript, we have ensured that **each compound is written in full the first time it appears**, followed immediately by its abbreviation in parentheses. After this point, only the approved abbreviation is used consistently throughout the manuscript.<br>The following modifications have been implemented:<br>• **Thymol (TM)**<br>• **Sodium azide (SA)**<br>• **Sodium lauryl sulfate (SLS)**<br>• **Phenyl azide (PA)**<br>• **Lauryl sulfate (LS)**<br>We have carefully checked **all sections**, including Abstract, Introduction, Methods, Results, Discussion, Figures, Tables, and

	Supplementary Files, to confirm that abbreviations are introduced correctly at first mention and used uniformly afterward.
The introduction is weak. The background leading to the development of hypothesis is lacking. Weak contextualization of the problem and the flow of discussion is also missing. It reads more like a descriptive summary of antimicrobial resistance rather than a tightly argued justification for why this specific combination of compounds and approaches were used in this study.	Thank you for this valuable comment. We have **substantially revised the Introduction** to improve the scientific narrative, strengthen the contextual background, and clearly connect the AMR problem to the selection of the compounds investigated in this study. The revised version now:<br>• Provides a **clear rationale** for choosing thymol (TM), sodium azide (SA), and sodium lauryl sulfate (SLS).<br>• Explains the **mechanistic reasoning** for including SA and SLS as comparator compounds.<br>• Builds a **logical progression** from AMR → biofilms → gaps in natural-compound research → justification for our integrated experimental–computational approach.<br>• Explicitly states the **central hypothesis** and research objectives.<br>These revisions address the reviewer's concerns regarding coherence, justification, and hypothesis development.
The novelty of the work is not highlighted.	We appreciate this observation. The Introduction and Discussion have been revised to **explicitly highlight the novelty** of our study. The novelty lies in the following aspects:<br>1. **Integrated approach:** To the best of our knowledge, this is the first study combining **in vitro antimicrobial and antibiofilm assays** with **300-ns molecular dynamics simulations, PCA/FEL analysis, and MM-GBSA** to investigate thymol and its mechanistic comparators.

	2. **Mechanistic benchmarking:** Previous thymol studies have not compared TM's behavior against **a respiration inhibitor (SA)** and **a membrane disruptor (SLS)**, offering unique mechanistic insights.<br>3. **Evaluation of structural analogs:** We introduce and evaluate **phenyl azide (PA)** and **lauryl sulfate (LS)** as simplified analogs of SA and SLS through computational modeling, an angle not explored in prior literature.<br>4. **Target-based mechanistic insight:** Our study evaluates TM's interaction with multiple microbial proteins simultaneously, providing a depth of mechanistic understanding unavailable in earlier works.<br>These points are now clearly stated in the revised manuscript to emphasize the originality of the work.
The antimicrobial activity of thymol is well studied. How this study is different from the previous studies should be justified. The authors can refer to the following articles-<br><br>a) https://www.frontiersin.org/journals/microbiology/articles/10.3389/fmicb.2020.01744/full<br>b) https://pmc.ncbi.nlm.nih.gov/articles/PMC9870694/	We thank the reviewer for suggesting these important references. Both articles have now been **cited and discussed** in the revised Introduction to clearly differentiate our work from previous thymol studies.<br>The differences are now explicitly stated:<br>• Previous studies primarily investigated **phenotypic outcomes** such as bacterial growth inhibition, membrane damage, or biofilm disruption.<br>• In contrast, our study integrates **long-timescale (300-ns) MD simulations**, PCA/FEL analysis, and MM-GBSA free energy calculations to validate protein–ligand interactions at an atomic level.

	• Earlier literature did not systematically compare thymol with **mechanistic control compounds**, limiting mechanistic interpretation.<br>• No previous work has explored **structural analogs (PA, LS)** to identify potential scaffolds for antimicrobial optimization.<br>These distinctions clearly position our study as a **more comprehensive and mechanistically rich investigation** relative to existing thymol studies.
6. It is not clear and why SA and SLS were chosen to benchmark TM? No standard drug/antibiotic was used in the experiment! justify their inclusion versus clinically relevant comparators.	We appreciate this important point. The justification has now been added and clarified in the revised Introduction.<br>SA and SLS were selected as **mechanistic comparators**, not therapeutic candidates, for the following reasons:<br>• **SA** is a well-defined **cytochrome oxidase inhibitor**.<br>• **SLS** is a well-known **membrane-disrupting surfactant**.<br>These compounds have **predictable, single-mode mechanisms**, enabling clear mechanistic benchmarking:<br>• TM vs **respiration inhibitor** (SA)<br>• TM vs **membrane disruptor** (SLS)<br>Using broad-spectrum antibiotics would not have allowed such mechanistic clarity because clinical antibiotics often act through **multiple simultaneous targets**, making comparative interpretation difficult. Thus, including SA and SLS gives a **clean mechanistic comparison**, rather than a potency ranking.<br><br>This justification is now explicitly stated in the revised manuscript.

The author used clinical isolate. Was it from microbial bank or direct from patient? The Ethical approval is not provided. If not required then explain why approval was not required.	The clinical isolates used in this study were **obtained from the microbial culture bank** of King Saud Hospital as **archived, de-identified diagnostic cultures**, not collected directly from patients. Since **no new samples were taken** and **no patient information was accessed**, the work did not involve human subjects. Therefore, **ethical approval was not required** according to institutional guidelines.
For statistical analysis, the number of replicates should be mentioned. Only biological replicate is acceptable but not the technical replicate.	Thank you for pointing this out. We have updated the **Materials and Methods** and **Figure Legends** to explicitly state the number of biological replicates used in each experiment. All antimicrobial, and antibiofilm assays were performed using **three independent biological replicates (n = 3)**.<br><br>Technical replicates were not used for statistical comparisons, in accordance with the reviewer's recommendation. This clarification is now included in the revised manuscript.
What was the final concentration of DMSO? State in the method.	We thank the reviewer for this helpful comment. The final DMSO concentrations used in all assays have now been clearly specified in the **Materials and Methods** section of the revised manuscript. As corrected after reviewing our experimental records, all working solutions were prepared such that the **final DMSO concentration did not exceed ≤10% v/v** in any assay. The updated details are as follows:<br>• **Disc diffusion assay:** Stock solutions were prepared in DMSO, and the volume applied ensured a **final diffusion disc exposure of 10% v/v DMSO**. A DMSO-only disc (10%)

	served as the negative control and showed no antimicrobial activity.<br>• **MIC/MBC assays:** The final concentration of DMSO in each microtiter well was controlled to **≤10% v/v**, with matching DMSO controls confirming no inhibitory effect.<br>• **MBIC/MBEC assays:** Test solutions were prepared such that the **final DMSO concentration remained ≤10% v/v**, and corresponding DMSO-only wells verified that DMSO did not interfere with biofilm formation or eradication.<br>These corrected concentrations (≤10% v/v) have now been explicitly incorporated into the Methods section for accuracy, reproducibility, and full transparency.
The quality of the figures should be improved substantially.	Thank you for highlighting this concern. All figures have now been regenerated at high resolution (600 dpi) with improved clarity, readability, and consistent formatting. Please note that the **conversion of the manuscript to PDF within the PLOS ONE submission system** can **sometimes compress or downscale images**, which may give the **impression of reduced quality**. However, the original source files submitted (Word documents and high-resolution TIFF images) retain full quality without any distortion. We kindly request you to refer to the original submission files for accurate figure resolution.
Protein 3D structure modeling method together with validation should be given if not in the manuscript but as supplement.	Thank you for the comment. The 3D model has now been clearly described, including the AlphaFold prediction source, pLDDT confidence scores, radius of gyration, and Ramachandran plot validation. All model-quality results are provided in **Supplementary Table S2** and **Supplementary Figures S1–S4**.

The conclusion is too broad. Focus the significant finding and the carry out message with future direction.	Thank you for the comment. The conclusion has been revised to focus specifically on the key findings of the study, the main takeaway message, and a concise future research direction. The updated version avoids broad general statements and now provides a clear, impactful summary aligned with the reviewer's recommendation.

Reviewer-4

The study's focus on repurposing thymol and eevaluating structural analogs (e.g., LS) as potential scaffolds is a meaningful contribution to antimicrobial drug discovery. However, the novelty is moderate thymol's antimicrobial potential is already well-documented, and the computational exploration of its binding to common microbial targetes has been reported in prior literature. The manuscript would benefit from a clearer articulation of what specifically is new about this study (e.g., the particular combination of targets, unique comparative approach, or mechanistic insight)	Thank you for this important comment. We agree that thymol's antimicrobial activity has been previously reported; however, our study offers several novel contributions that go beyond existing literature. Specifically:<br>• **Integrated mechanistic benchmarking:** This is the first study to directly compare thymol with mechanistic controls—sodium azide (respiration inhibitor) and sodium lauryl sulfate (membrane disruptor)—to dissect its mode of action relative to defined mechanisms.<br>• **Deep structural validation using long-timescale simulations:** Prior thymol studies have not incorporated **300 ns MD simulations, PCA/FEL analysis, and MM-GBSA energy decomposition** across multiple microbial targets simultaneously.<br>• **Evaluation of structural analogs (PA and LS):** We introduce and evaluate simplified analogs of SA and SLS as **computational scaffolds**, which has not been explored before.<br>• **Multi-target mechanistic insight:** Unlike earlier reports focused on single targets, our study examines thymol's interactions across a **broader panel of essential microbial proteins**, revealing target-specific stability profiles supported by atomistic simulations.

	We have now explicitly articulated these points in the Introduction and Discussion to clearly highlight the novelty and unique mechanistic depth of the present work.
The in-vitro assays (MIC, MBC, and biofilm inhibition) are described adequately, but it is not always clear how many biological or technical replicates were performed. Including these details would enhance reproducibility.	Thank you for pointing this out. We have now clearly stated the number of replicates used in all in-vitro experiments. All MIC, MBC, and biofilm inhibition assays were performed using **three independent biological replicates (n = 3)**, and technical replicates were not used for statistical comparisons. These details have been added to the Materials and Methods section and the corresponding figure legends to ensure full reproducibility.
The selection of control agents and bacterial strains seems logical, though some justification for their choice would help (e.g., why these particular MDR species were prioritized).	Thank you for this valuable comment. The selection of control agents and bacterial strains has now been clearly justified in the manuscript. The MDR strains used in this study were chosen because they represent **clinically significant pathogens frequently encountered in our regional hospital setting**, known for their **high resistance profiles, strong biofilm-forming ability, and relevance to persistent hospital-acquired infections**. These characteristics make them suitable and meaningful models for evaluating both antimicrobial and antibiofilm performance.<br><br>The control agents—sodium azide (SA) and sodium lauryl sulfate (SLS)—were intentionally selected as **mechanistic comparators** rather than therapeutic standards. SA functions as a cytochrome oxidase inhibitor, and SLS as a membrane-disrupting surfactant, enabling us to benchmark thymol's activity against compounds with **well-defined and distinct mechanisms of action**. This provides mechanistic clarity that would not be achievable using conventional antibiotics, which often have multifaceted modes of action.

The computational workflow (molecular docking MD simulations, MM-GBSA, PCA/FEL) is methodologically robust. Yet, the assumed correlation between ΔG and IC_{50} could be discussed more cautiously; this relationship is non-linear and depends heavily on experimental context.	Thank you for this insightful comment. We agree that the relationship between predicted binding free energy (ΔG) and experimental IC_{50} values is not strictly linear and can vary significantly depending on assay conditions, kinetic factors, and target-specific dynamics. In the revised manuscript, we have now added a cautionary clarification noting that ΔG- and Ki-based estimations provide relative comparative trends, not absolute potency predictions. We have emphasized that these computational values should be interpreted as supportive indicators rather than direct substitutes for experimentally determined IC_{50}, underscoring the need for future *in vitro* validation.
While 300-ns MD simulations are impressive, the analysis would be strengthened by including convergence checks such as clustering or RMSD stability analysis beyond PCA/FEL plots.	Thank you for this valuable suggestion. We agree that convergence assessment is important for validating MD simulation reliability. In the revised manuscript, we have now included RMSD stability evaluation across the 300 ns trajectory and clarified that the systems reached stable equilibrium after the initial fluctuations. Although full clustering analysis was not performed, the RMSD, Rg, SASA, and H-bond profiles collectively indicate well-converged trajectories. This information has been added to the MD Results subsection and referenced accordingly.
The integration of in-vitro and in-silico data is one of the paper's strongest features, clearly linking molecular behavior with biological activity. However, the presentation could be streamlined: several figures and tables seem to repeat similar information (e.g., multiple mentions of binding energies or RMSD trends). Condensing these could improve readability.	Thank you for this constructive comment. We agree that multiple MD and docking figures conveyed overlapping information. To improve readability and streamline presentation, we have reorganized the figures as follows:<br>1. **Only representative summary figures** are retained in the main manuscript.

	2. **All repetitive or per-complex MD plots** have been moved to the Supplementary File.<br>3. The total number of main-text figures is now **13**, as recommended by the other reviewer.<br>This restructuring maintains all scientific details while presenting a clearer and more concise narrative.
The discussion of SA and SLS is appropriately cautious, acknowledging their toxicity and poor pharmacokinetics. Still, a deeper reflection on why these compounds were chosen beyond being "comparative controls" would clarify their scientific role	We thank the reviewer for this important observation. To clarify the scientific basis for selecting sodium azide (SA) and sodium lauryl sulfate (SLS), we have added a dedicated subsection in the Introduction titled ***"Rationale for Using Sodium Azide (SA) and Sodium Lauryl Sulfate (SLS)"*** This section explains that SA and SLS were chosen as mechanistic controls representing two well-defined antimicrobial mechanisms—respiration inhibition and membrane disruption, respectively. This provides a mechanistic benchmark to contextualize thymol's behavior and avoids confounding interpretations associated with broad-spectrum antibiotics. We also highlight that their single-mode mechanisms offer clean baselines that help distinguish true target-specific effects of TM, PA, and LS. The revised text now clarifies their scientific role beyond being comparative controls.
Nonetheless, the analysis occasionally mixes predicted and literature-derived toxicities without clearly separating computational data from empirical evidence. Labeling which parameters were experimentally validated versus purely predicted would enhance transparency.	Thank you for pointing this out. We clarify that all toxicity and pharmacokinetic assessments in this study were obtained **exclusively from *in silico* predictions using ADMETlab 3.0**, and no *in vitro* toxicity experiments were performed. In the revised manuscript, we now explicitly state that all toxicity values are purely computational predictions. We have also updated the sections.

The language could be tightened certain sections repeat the same point (e.g., TM's superiority) multiple times.	We thank the reviewer for this helpful comment. We have carefully revised the manuscript to tighten the language and reduce repetition regarding TM's superiority. In particular, we streamlined the Results and Discussion opening, removed/softened mini-conclusions in the MIC/MBIC/MBEC and ADMET sections, and kept a single overarching statement of TM's overall superiority in the Abstract and in the Conclusion only. We believe this addresses the concern and improves readability.
The connection to clinical translation could be expanded: what specific formulations or combination therapies are most feasible for TM?	Thank you for this constructive suggestion. We agree that highlighting the clinically feasible formulations and combination strategies for thymol (TM) would strengthen the translational perspective of the study. In the revised manuscript, we have added a brief section discussing the most promising formulation approaches (e.g., nanoemulsions, liposomes, hydrogels) and potential synergistic combinations with antibiotics commonly used for MDR infections.
The paper would benefit from a graphical summary or schematic showing the overall workflow (in-vitro testing docking,MD, ADMET, outcome).	We thank the reviewer for this valuable suggestion. We would like to clarify that a **graphical workflow summary was already included in our original submission** as the graphical abstract . This figure illustrates the complete study pipeline, including:<br>• Test compounds<br>• In-vitro antimicrobial and antibiofilm assays<br>• In-silico docking and molecular dynamics simulations<br>• Toxicological/ADMET evaluation<br>• Integrated mechanistic outcome<br>We appreciate the reviewer's suggestion, which helped us improve visibility of this component.

Technical terms are used correctly, though some sentences are overly long or complex. Simplifying these would make the text more reader-friendly.	We thank the reviewer for this helpful observation. In response, we have thoroughly revised the manuscript to improve clarity and readability. Several long or complex sentences have been simplified, shortened, or restructured to ensure smoother flow and better comprehension. These modifications were applied throughout the **Introduction**, **Results and Discussion**, and **Conclusion** sections.<br>All technical terms have been retained accurately, but the surrounding text has been made more concise and reader-friendly while preserving scientific precision.
Figures (especially PCA/FEL plots) should include better labeling and resolution, with consistent font sizes and color schemes to enhance readability.	We thank the reviewer for this valuable suggestion. In response, we have improved all PCA and FEL figures and moved them to the Supplementary Data file to enhance clarity and consistency. Specifically:<br>• All figures were **regenerated at high resolution (600 dpi)**.<br>• **Font sizes** for axis labels, legends, and titles were standardized across all figures.<br>• **Color schemes** were unified to ensure consistent visualization of conformational clusters and energy minima.<br>• **Backgrounds and layout** were adjusted to maintain uniform, professional formatting throughout.<br>The revised figures are now included in the updated manuscript and supplementary materials.
Explicitly state what new insight this study provides beyond existing thymol literature.	This comment has been addressed by explicitly articulating the novel insights and unique contributions of our study in the Introduction and Discussion sections.

Include replicate numbers, control information, and statistical tests.	We thank the reviewer for this important comment. We have now included full details regarding replicate numbers, control conditions, and statistical analyses. Specifically:<br>• All experiments were conducted using **three biological replicates (n = 3)**.<br>• Appropriate **negative controls (DMSO-only)** were included for all assays, and their lack of antimicrobial effect was confirmed.<br>• Statistical analyses and reporting of **mean ± SD** have been added throughout the Methods, Results, and Figure Legends.<br>These revisions improve the methodological transparency and reproducibility of the study.
Address limitations of Ki ≈ Kd assumptions and possible docking bias.	We thank the reviewer for this important point. We have now added a statement in the Discussion addressing the limitations associated with assuming $K_i \approx K_d$ and the inherent biases of molecular docking.<br>Specifically, we acknowledge that:<br>• **$K_i \approx K_d$ is an approximation** used in computational docking because most scoring functions estimate binding affinity based on enthalpic contributions and do not directly compute kinetic rate constants. Therefore, these values should be interpreted as **relative indicators**, not absolute experimental Ki or Kd values.<br>• **Docking has intrinsic biases**, including:<br>○ rigid-receptor assumptions<br>○ limited sampling of protein flexibility

	◦ scoring function inaccuracies<br>◦ potential overestimation of hydrogen bonds and hydrophobic contacts<br>• To mitigate these biases, we complemented docking with **300 ns MD simulations**, RMSD/RMSF/Rg/SASA analysis, **MM-GBSA free-energy calculations**, and PCA/FEL conformational sampling, providing a more reliable assessment of binding stability.<br>This limitation note has now been included in the revised **Discussion** section.
Distinguish predicted vs. empirical ADMET findings.	We clarify that all toxicity and pharmacokinetic assessments in this study were obtained exclusively from *in silico* predictions using ADMETlab 3.0, and no *in vitro* toxicity experiments were performed. In the revised manuscript, we now explicitly state that all toxicity values are purely computational predictions.
Improve figure quality and legends and remove the back ground colors from the figures (must needed).	We have improved the figure quality and updated the legends. The background colors of multiple figures have been removed, and all visual elements are now presented with a clean white background as per the reviewer's suggestion.
