# Supplementary material for: Antimicrobial and antibiofilm evaluation of thymol, sodium azide, and sodium lauryl sulfate against multidrug-resistant pathogens: An integrated experimental and computational study
Source: PLoS One. 2026 Apr 7;21(4):e0345977. doi: 10.1371/journal.pone.0345977 (PMC13056186; doi:10.1371/journal.pone.0345977)
Supplement: Attachment — Submitted filename: Response to Reviewer-5.pdf [file pone.0345977.s037.pdf]

Response to Reviewer-5's Comments

We sincerely thank the Reviewer for their thorough evaluation of our manuscript and for the detailed, constructive comments provided. We highly appreciate the time and expertise invested in this review. The suggestions have helped us improve the clarity, rigor, and presentation of the manuscript.

Please note that the manuscript has already undergone **multiple rounds of peer review**, and the present revision focuses on **clarifications, methodological transparency, textual refinement, and figure improvement**, without repeating experimental or computational investigations. All feasible corrections have been implemented carefully while preserving the scientific integrity and flow of the work.

Our point-by-point responses are provided below.

All figures are poorly presented.	We thank the reviewer for highlighting this issue. All figures have now been **regenerated at high resolution (600 dpi)** with improved layout, font size, contrast, and consistency. Figure captions were also refined for clarity and completeness.<br><br>We note that **PDF conversion within the submission system may compress images**; however, **original high-resolution source files (TIFF/PNG)** are provided in the **submission** and **retain full quality.**
Comment #0: The manuscript must be revised, where some typos and grammatical errors were observed.	The entire manuscript has been **thoroughly proofread** and linguistically refined. Typographical errors, grammatical inconsistencies, and stylistic redundancies were corrected throughout the text.

Comment #1: The introduction provides a general context for the study. However, it does not explain the premises of the work, nor does it provide a more specific rationale for the study. Additionally, the results should not be presented in the introduction section.	We thank the reviewer for this constructive comment. The Introduction has been substantially revised to clearly articulate the premises and rationale of the study by explicitly identifying the knowledge gaps motivating the work, clarifying the mechanistic basis for selecting thymol and the comparator compounds, and clearly stating the study objectives and working hypotheses. In addition, all results-related statements have been removed from the Introduction and are now presented exclusively in the Results and Discussion section, thereby improving conceptual clarity and adherence to standard manuscript structure.
Comment #2: Could the authors clarify the rationale for selecting this older PDB entry, despite the availability of more recent structures with improved resolution?	We thank the reviewer for this comment. The rationale for selecting the PDB structures has now been explicitly clarified in the Methods section. Although newer structures with higher nominal resolution are available for some targets, the selected PDB entries were chosen based on the presence of co-crystallized ligands, well-defined and functionally relevant active-site conformations, and overall structural completeness, particularly in regions surrounding the binding pocket. Where applicable, newer structures were evaluated; however, the selected entries provided greater methodological consistency for comparative docking and molecular dynamics analyses across multiple targets, which was central to the study design.
Comment #3: 1000 ps is not sufficient for the equilibration stage of the investigated complexes nowadays. It should be at least 10 ns.	We thank the reviewer for this important observation. We clarify that the reported 1000 ps corresponds to each equilibration phase, comprising 1000 ps under NVT conditions followed by 1000 ps under NPT conditions prior to the 300 ns production simulations. The adequacy of equilibration was verified by monitoring

	backbone RMSD stabilization, as well as convergence of temperature, pressure, and system density before initiating production runs. This clarification has now been explicitly added to the Molecular Dynamics simulation protocol section of the manuscript.
Comment #4: The authors claimed that "the ADMET evaluation was conducted using SwissADME to assess the drug-likeness properties of the selected compounds". However, there is a big difference between ADMET and drug likeness properties. Please revise and correct.	We thank the reviewer for this important clarification. The manuscript has been revised to clearly distinguish between **ADMET predictions** and **drug-likeness considerations**. ADMETlab 3.0 is now described exclusively for predicting absorption, distribution, metabolism, excretion, and toxicity endpoints, while drug-likeness is discussed separately as supportive physicochemical context and not as a substitute for full ADMET evaluation. This revision removes the previous conceptual overlap and ensures accurate and consistent terminology throughout the manuscript.
Comment #5: Full technical details regarding molecular docking and molecular dynamics simulations should be provided in the revised manuscript.	We thank the reviewer for this comment. The revised manuscript now provides full and explicit technical details for both molecular docking and molecular dynamics simulations in the Methods section. These include software versions, force fields, solvation models, ion concentrations, cutoff values, thermostats and barostats, equilibration and production protocols, trajectory sampling parameters, validation procedures, and post-simulation analysis workflows (RMSD, RMSF, MM-GBSA, PCA, and FEL). Collectively, these additions ensure methodological transparency, reproducibility, and compliance with current computational modeling standards.

Comment #6: How did the authors compute the atomic charges of the investigated compounds prior to molecular dynamics simulations?	We thank the reviewer for this comment. The manuscript has been revised to clarify that ligand parameters and atomic partial charges were generated using CHARMM-compatible force-field tools based on the CHARMM General Force Field (CGenFF), ensuring full consistency with the CHARMM36m framework employed for molecular dynamics simulations. The exact procedure is now clearly described in the revised manuscript.
Comment #7: How did the authors compute the atomic charges of the investigated compounds prior to molecular docking computations?	We thank the reviewer for this question. Atomic partial charges for molecular docking were assigned automatically during ligand preparation using the internal charge assignment protocol of the docking software, following its standard and validated workflow. These charges were used exclusively for docking calculations and are independent of the force-field–based charges employed later for molecular dynamics simulations. This clarification has now been explicitly added to the revised manuscript.
Comment #8: Figures 7 to 12 should be combined into one Figure.	We thank the reviewer for this suggestion. Figures **7–12 have been reorganized and combined** to improve clarity and avoid overcrowding. Specifically, the interaction analyses were grouped **ligand-wise** into three multi-panel figures: **Fig. 4 (TM)**, **Fig. 5 (PA)**, and **Fig. 6 (LS)**, each presenting combined 2D and 3D interaction views across the respective target proteins. This restructuring reduces redundancy while preserving clear visualization of individual ligand–target interactions.
Comment #9: Molecular interactions of the predicted docking poses of the identified analogs should be detailed in the revised manuscript.	We thank the reviewer for this suggestion. The revised manuscript now provides a detailed characterization of the molecular interactions associated with the predicted docking poses of all identified analogs. A comprehensive interaction table has been

	added, and the Results and Discussion sections have been expanded to describe hydrogen bonding, hydrophobic contacts, π–π stacking interactions, salt bridges, and water-mediated hydrogen bonds for each protein–ligand complex. These additions ensure a complete and transparent presentation of the docking interaction profiles.
Comment #10: Post-MD calculations should include binding energy per frame, center-of-mass, and hydrogen bond analyses..., etc.	We thank the reviewer for this suggestion. The revised manuscript already includes extensive post-MD analyses, including hydrogen-bond analysis, RMSD, RMSF, Rg, SASA, MM-GBSA binding free energy calculations, PCA, and FEL mapping, which together comprehensively characterize binding stability and conformational behavior. Additional analyses such as center-of-mass tracking and per-frame binding energy decomposition were not included to avoid redundancy, and this rationale has now been explicitly clarified in the manuscript.
Comment #11: Reference style must be revised. There are versatile omitted details.	All references have been **reformatted and checked** for completeness and consistency according to journal guidelines.
Comment #12: The conclusion section must be rewritten to be more informative and unveil the most beneficial outcomes.	We thank the reviewer for this valuable comment. The Conclusion section has been fully rewritten to provide a clearer and more informative synthesis of the study's key experimental and computational findings. The revised Conclusion now emphasizes mechanistic insights derived from docking and long-timescale MD simulations, highlights the translational relevance of thymol as a promising antimicrobial scaffold, and clearly outlines the limitations of the current study along with future research directions. These revisions strengthen the interpretative value and practical relevance of the conclusions.

Comment #13: All docking scores and binding energies should be in one decimal unit.	Thanks for your valuable comment. All docking scores and MM-GBSA binding energies have been uniformly rounded to one decimal place for consistency.
Comment #14: Did the authors investigate the protonation state of the titrable amino acids before docking?	We thank the reviewer for this important question. Yes, protonation states of titratable amino acid residues were considered during protein preparation. All proteins were protonated at physiological pH (~7.4) using the MOE QuickPrep protocol prior to docking, ensuring appropriate ionization of titratable residues (Asp, Glu, His, Lys, and Arg). This clarification has now been explicitly added to the Molecular Docking Simulation section of the revised manuscript.
Comment #15: Did the authors investigate the protonation state of the ligands before docking computations?	Yes. Ligands were prepared with appropriate protonation states at physiological pH (~7.4) using the MOE QuickPrep/ligand preparation workflow, which automatically assigns protonation and ionization states consistent with standard docking protocols. This clarification has now been explicitly added to the Methods section.
Comment #16: Full technique details regarding molecular docking computations should be considered in the revised manuscript, such as the grid spacing value.	We thank the reviewer for this valuable suggestion. As the Molecular Operating Environment (MOE) employs a placement- and scoring-function–based docking strategy rather than an explicit grid-spacing parameter, the docking methodology has been clarified accordingly. The revised manuscript now explicitly describes the binding-site definition, Triangle Matcher placement strategy, induced-fit refinement, London dG scoring and pose-selection criteria used in MOE, thereby ensuring full transparency and reproducibility of the docking computations.

Comment #17: The reference of the utilized pdb code should be cited in the revised manuscript.	We thank the reviewer for this comment. All PDB entries utilized in this study are now explicitly cited in the revised manuscript, including references to the original structure-determination publications and the Protein Data Bank database, thereby ensuring proper attribution and full traceability of the structural data.
Comment #18: Did the authors perform equilibration for the investigated complexes prior to executing molecular dynamics simulations?	We thank the reviewer for this comment. Yes, all systems were subjected to a standard equilibration protocol prior to production molecular dynamics simulations. Each protein–ligand complex underwent energy minimization followed by sequential NVT and NPT equilibration phases before initiating the 300 ns production runs. This equilibration procedure has now been explicitly clarified and emphasized in the revised Molecular Dynamics methodology section.

Final Remark

We sincerely thank the Reviewer for their valuable feedback. We believe that the revisions have substantially strengthened the manuscript in terms of clarity, methodological transparency, and scientific rigor—while preserving the validated experimental and computational framework.
