# Supplementary material for: Antimicrobial and antibiofilm evaluation of thymol, sodium azide, and sodium lauryl sulfate against multidrug-resistant pathogens: An integrated experimental and computational study
Source: PLoS One. 2026 Apr 7;21(4):e0345977. doi: 10.1371/journal.pone.0345977 (PMC13056186; doi:10.1371/journal.pone.0345977)
Supplement: Attachment — Submitted filename: Reviewers Comment for PONE-D-25-53573R1.docx [file pone.0345977.s035.docx]

**Reviewer’s Comment**

**Manuscript Number:** PONE-D-25-53573R1

**Title:** Antimicrobial and Antibiofilm Evaluation of Thymol, Sodium Azide, and Sodium Lauryl Sulfate Against Multidrug-Resistant Pathogens: An Integrated Experimental and Computational Study

This manuscript presents a timely and methodologically robust investigation into the repurposing potential of three compounds against multidrug-resistant pathogens. The study effectively bridges experimental microbiology with advanced computational modeling, addressing a critical need for novel antimicrobial strategies, particularly against biofilms. The work aligns well with PLOS ONE's scope of publishing rigorous, multidisciplinary scientific research.

**Strengths Supporting Acceptance:**

1. **Significant and Relevant Topic:** The research tackles the pressing global health challenge of antimicrobial resistance (AMR) and biofilm-associated infections, a core interest area for the scientific and medical community.
2. **Comprehensive and Integrated Methodology:** The combination of in vitro assays (MIC, MBC, MBIC, MBEC) across a broad panel of clinically relevant bacterial and fungal strains with extensive in silico analyses (molecular docking, 300 ns MD simulations, MM-GBSA, PCA/FEL, ADMET) represents a notable strength. This multi-pronged approach provides a more complete picture of the compounds' potential than either approach alone.
3. **Clear and Compelling Findings:** The abstract succinctly presents differentiated roles for the tested agents:
   - **Thymol (TM)** is identified as a strong, broad-spectrum repurposing candidate with dual antimicrobial/antibiofilm activity and a favorable predicted safety profile.
   - The study intelligently uses **SA and SLS** as mechanistic benchmarks, providing context for the novel findings.
   - The in silico exploration of **non-ionic analogs (PA, LS)** as optimized scaffolds, particularly the high predicted affinity of LS for LpxC, generates a valuable hypothesis for future drug development.
4. **Rigorous Computational Analysis:** The use of long-timescale MD simulations (300 ns), binding free energy calculations (MM-GBSA), and conformational analysis (PCA/FEL) moves beyond simple docking to provide credible insights into binding stability and dynamics, strengthening the mechanistic hypotheses.
5. **Translational Perspective:** The focus on "repurposing potential" and the inclusion of in silico ADMET profiling directly address the practical hurdles in antimicrobial development, increasing the translational relevance of the work.

**Overall Assessment:**

I recommend an acceptance because this is a well-conceived and expertly executed study that makes a valuable contribution to the field of AMR research. The integration of complementary techniques strengthens the conclusions and provides a clear pathway for future work. The manuscript is likely to be of broad interest to microbiologists, pharmacologists, and computational biologists.
